# The executive function of bilingual and monolingual children: A technical efficiency approach

**DOI:** 10.3758/s13428-021-01658-7

**Published:** 2021-09-10

**Authors:** Athanasia Papastergiou, Vasileios Pappas, Eirini Sanoudaki

**Affiliations:** 1 School of Languages, Literatures, Linguistics and Media, Bangor University, Bangor, UK; 2 Kent Business School, University of Kent, Kent, UK

**Keywords:** Bilinguals, Technical efficiency, DEA, Executive function, k-means, Bootstrap

## Abstract

This paper introduces a novel approach to evaluate performance in the executive functioning skills of bilingual and monolingual children. This approach targets method- and analysis-specific issues in the field, which has reached an impasse (Antoniou et al., 2021). This study moves beyond the traditional approach towards bilingualism by using an array of executive functioning tasks and frontier methodologies, which allow us to jointly consider multiple tasks and metrics in a new measure; *technical efficiency (TE)*. We use a data envelopment analysis technique to estimate TE for a sample of 32 Greek–English bilingual and 38 Greek monolingual children. In a second stage, we compare the TE of the groups using an ANCOVA, a bootstrap regression, and a k-means nearest-neighbour technique, while controlling for a range of background variables. Results show that bilinguals have superior TE compared to their monolingual counterparts, being around 6.5% more efficient. Robustness tests reveal that TE yields similar results to the more complex conventional MANCOVA analyses, while utilising information in a more efficient way. By using the TE approach on a relevant existing dataset, we further highlight TE’s advantages compared to conventional analyses; not only does TE use a single measure, instead of two principal components, but it also allows more group observations as it accounts for differences between the groups by construction.

## Introduction

A large strand of the empirical research on bilingualism focuses on the comparative performance of bilingual and monolingual populations with regards to executive function.^1^ On the one hand, a number of studies suggest that bilinguals outperform monolinguals on executive function tasks, in a so-called “bilingual advantage” (Bialystok, 2001; Bialystok et al., 2004, 2006; Bialystok & Martin, 2004; Calvo & Bialystok, 2014; Emmorey et al., 2008). On the other hand, there is increasing evidence that the “bilingual advantage” may not be as universal as originally suggested. In particular, the bilingual advantage may be confined within particular age ranges, such as preschool children or older adults (Bialystok, 2017; Hilchey & Klein, 2011), or specific subcategories of executive function; thus prohibiting generalisations (Bialystok et al., 2009).

This lack of consensus in the literature may be attributed to several factors, broadly grouped into two categories; method-specific and analysis-specific. Method-specific differences comprise the particulars of executive function tasks, such as the administered task, and whether the investigated quantity is the accuracy and/or the reaction time. Some of the executive function tasks that have been used include the dimensional change card sort (DCCS) task (Zelazo et al., 1996), the Children’s Embedded Figures Task (Bialystok & Shapero, 2005), the flanker task (Calvo & Bialystok, 2014; de Abreu et al., 2012; Kapa & Colombo, 2013; Yang et al., 2011), the attentional network task (ANT) (Antón et al., 2014; Poarch & van Hell, 2012; Yang et al., 2011), the Simon task (Poarch & van Hell, 2012), the Stroop task (Antón et al., 2014; Poulin-Dubois et al., 2011) and the Multilocation task (Poulin-Dubois et al., 2011). The majority of studies report a single test, while Poulin-Dubois et al. (2011) is one of the few that report five, which, not surprisingly, lead to different conclusions. Analysis-specific differences comprise variations in the data cleaning, and subsequent analyses; most notably controlling for participant-specific characteristics. As most studies in this field feature small samples, certain limitations are, perhaps, unavoidable. For example, controlling for (or matching on) children’s grade (or age) and socio-economic status (SES), might exclude performance differences attributed to vocabulary and grammar skill differences in both languages, to name but a few. The need to control for an extensive array of indicators has been highlighted in Paap and Greenberg (2013) within this context, and within Stuart (2010) in a broader sense.^2^

In this paper, we aim to address both method-specific and analysis-specific issues, by presenting a novel approach that relies on the frontier methodology that measures the relative efficiency of a decision-making unit (DMU) compared to the best practice, in what is termed as technical efficiency. This is a flexible methodology; due to it being a non-parametric, linear programming technique, it does not rely on distributional assumptions and is not computationally intensive. We apply this methodology in the context of executive function performance evaluation of Greek–English bilingual and Greek monolingual children, while using an extended array of executive function tasks and metrics that are in line with the related literature in this field.

## Bilingualism and executive control: mechanisms and challenges

The executive function system is a domain-general cognitive system, vital for the flexibility and regulation of cognition and goal-directed behaviour (Best & Miller, 2010). It is referred to as the most crucial cognitive achievement in early childhood (Abutalebi & Green, 2007; Bialystok et al., 2009; Del Maschio et al., 2018). Children gradually master the ability to control attention, inhibit distraction, monitor sets of stimuli, expand working memory, and shift between tasks. The bilingual advantage refers to the superior performance of bilinguals in tasks that seem to require executive processing, which is the ability to monitor goal-setting cues, to switch attention to goal-relevant sources of information, and to inhibit those that are irrelevant or competing (Bialystok & Martin, 2004; Costa et al., 2008; Emmorey et al., 2008). These advantages in executive functions of bilinguals are thought to be linked to the need for this population to manage multiple languages and continuously monitor the appropriate language for each communicative situation (Bialystok, 2009). More specifically, bilinguals need to select the right language for each circumstance, attend to cues in order to select the right language, select the suitable lexicon and at the same time suppress the interference of the other language/s generating general executive function advantages (Bialystok, 2017; Green, 1998). This consistent exposure to a context where higher-level cognitive function is constantly required may contribute to advanced cognitive performance. High-level cognition is theorized to be required, such as working memory to maintain and manipulate information and inhibitory control to block or ignore competing information internally or from the environment (e.g., irrelevant words). This high-level cognition has been purported to contribute to across-the-board cognitive performance gains, dubbed as “bilingual advantage”.

Many studies have focused on childhood bilingualism and executive control, documenting that bilingual children outperform their monolingual cohorts on executive functioning tasks (Adesope et al., 2010; Bialystok, 2017), including selective attention (Bialystok, 2001), cognitive flexibility (Poulin-Dubois et al., 2011) and working memory (Morales et al., 2013). However, several other studies have not detected a bilingual effect on the executive function domain (Antón et al., 2014; Gathercole et al., 2014; Valian, 2015).

Paap (2018) and Paap et al. (2014) highlight a number of reasons that may be driving the results towards a “bilingual advantage”. Small samples might be one of the caveats, as studies with larger sample sizes tend to report no significant differences between bilinguals/monolinguals (Paap et al., 2015). Several studies featuring large datasets (Antón et al., 2014; Duñabeitia et al., 2014; Gathercole et al., 2014; Paap et al., 2014, 2017; Paap & Greenberg, 2013) reject the existence of a bilingual advantage.^3^ In addition, a series of meta-analyses suggest that the bilingual advantage is either of very small magnitude (De Bruin et al., 2015; Grundy & Timmer, 2017) or non-existent (Donnelly, 2016; Lehtonen et al., 2018). Population-specific differences including variations in the bilingualism definition (Namazi & Thordardottir, 2010), differences/similarities in the languages the bilinguals manage (Bialystok, 2017; Yang et al., 2011), the switching intensity and/or frequency between the two languages (Baddeley, 2003) and cultural differences (Paap, 2018) may also affect the results.

Often the statistical analysis employs AN(C)OVA designs (Calvo & Bialystok, 2014; Poulin-Dubois et al., 2011), while regression techniques (Cox et al., 2016; Crivello et al., 2016), and propensity score matching (Tare & Linck, 2011) approaches tend to be limited. Over-reliance on ANCOVA and similar techniques is not a panacea, and underlying assumptions need to be checked thoroughly. In particular, Paap (2018) critiques how the correlation between the treatment variable and the control variables can be responsible for the appearance of a spurious bilingual advantage. For example, participation in team sports and musical dexterities have been linked to superior executive function (Paap et al., 2018; Paap & Greenberg, 2013; Valian, 2015).^4^ Team sports performance is positively correlated with executive function; the relationship being more pronounced for professional sports at high levels of competition (Paap et al., 2018; Vestberg et al., 2012). Valian (2015) observed that in studies with bilingual and monolingual children, the participants might get different amounts of exercise or might have experienced some other beneficial experience (e.g., musical training) influencing their executive functioning skills.

Inappropriate controlling strategies may also play a role in whether a bilingual advantage is detected. While it is common practice to match on age and SES, less-clear guidance exists for non-verbal intellectual ability and/or language skills. As non-verbal intellectual ability is correlated with particular aspects of executive function (e.g., working memory) (Friedman et al., 2006), matching groups on non-verbal intellectual ability may mitigate the bilingual advantage (Lehtonen et al., 2018). Bilingual language skills may be inferior to monolinguals (Calvo & Bialystok, 2014; Lehtonen et al., 2018); hence both appropriately assessing language skills to ensure a level playing field and matching are imperative (Bialystok et al., 2008).

Differences in the particulars of executive function tasks, such as the administered task and subsequent modifications, whether quantity of interest is the accuracy and/or the response time, may also be affecting the results. Miyake et al. (2000) classify executive function into updating, switching, and inhibition subcategories using latent factor analysis. Subsequent research attempts to proxy these subcategories using certain measures (e.g., antisaccade tasks for inhibition). As highlighted in Paap and Greenberg (2013), studies often use a single task for each executive function component, while De Bruin et al. (2015) find that studies in support of a bilingual advantage tend to report fewer tasks. Proxying for any of the subcategories of executive function relies on the implicit assumption that all proxies for, say, inhibitory control would: i) lead to the same conclusion; ii) be correlated with each other. Failure to observe both conditions suggests that no compelling evidence with regards to the bilinguals’ performance may be reached, as argued in Paap and Greenberg (2013). As such, puzzling results may be reached with a subset of measures suggesting a bilingual advantage, while others not concurring with these (Poulin-Dubois et al., 2011; Tao et al., 2011). This has been identified as the “task impurity problem” where accurate measurement of particular domains of executive function suffers from the fact that the multitude of measures do not tap into the same cognitive processes, besides reported reliability and validity concerns (Lehtonen et al., 2018; Paap & Sawi, 2016). For inhibition alone, a variety of tasks have been used including the antisaccade task (Paap et al., 2014; Paap & Greenberg, 2013), flanker task (Calvo & Bialystok, 2014; de Abreu et al., 2012; Kapa & Colombo, 2013; Paap et al., 2014; Von Bastian et al., 2016; Yang et al., 2011), Simon task (Antoniou et al., 2016; Gathercole et al., 2014; Paap et al., 2014; Paap & Greenberg, 2013; Poarch & van Hell, 2012; Von Bastian et al., 2016), Stroop (Calvo & Bialystok, 2014; Duñabeitia et al., 2014; Poulin-Dubois et al., 2011; Von Bastian et al., 2016), ANT (Antón et al., 2014; Paap & Greenberg, 2013; Poarch & van Hell, 2012; Yang et al., 2011). The present study aims to address these issues by using a comprehensive approach utilising information from multiple subcategories of executive function as per the Miyake et al. (2000) classification.

### The current study

In this paper, we present a novel methodology that accounts for the extended array of executive function tasks and metrics. Our method relies on the frontier methodology that measures the relative efficiency of a decision-making unit (DMU) compared to the best practice, in what is termed as *technical efficiency*. The technique is well-established in the areas of banking, economics, finance, transportation and management (Berger & Humphrey, 1997; Berger & Mester, 1997; Chen et al., 2015). Chen et al. (2015) verify that efficiency analysis is scarce in the management literature, even though its applicability is justified on a number of occasions. Within the fields of linguistics and psychology, efficiency applications are non-existent. We could not find any study using the frontier methodology in any of the highest-ranked journals (*Cognition, Psychological Research, Psychonomic Bulletin & Review, Journal of Memory and Language, Psychological Methods, Psychometrika, Psychological Science, The British Journal of Mathematical and Statistical Psychology, Current Directions in Psychological Science*) despite the fact that issues faced by researchers in these areas are not markedly different from the areas where the efficiency methodology has been successfully used.^5^

Technical efficiency allows the researcher to jointly examine multiple executive function tasks, while taking into consideration both the accuracy and the response time of the participant in each task. As such technical efficiency may be viewed as a special case of principal component analysis (PCA) technique, however, the two techniques are markedly different. Like PCA, technical efficiency can handle a large number of executive function tasks (identified as “outputs”). Conversely to PCA, technical efficiency accommodates, by construction, factors that may affect the performance in executive function tasks, such as age and non-verbal intellectual ability to name but a few (identified as “inputs”). As technical efficiency is a single variable it dispenses with the need of PCA to interpret the retained factors. Due to its non-parametric nature, it does not impose any distributional assumptions on the data, while as it does not rely on correlations between variables, it can accommodate cases where executive function tasks show low correlation (see Paap and Greenberg (2013) and references therein). For these advantages, we opt for a frontier approach in this paper.

We contribute to the literature in four distinct ways. First, we introduce technical efficiency methodology and highlight the similarities and advantages of the technique to alternative ones that are popular in this field. We provide an application of this technique to an unstudied dataset on the executive functions of Greek–English bilingual and Greek monolingual young children. In addition, we employ an alternative dataset which we analyse with our technical efficiency approach. Second, we contribute to the monolingual/bilingual literature by comparing the executive function scores of bilingual and Greek monolingual young children. Our executive function tests span attention, working memory, and inhibition; hence allowing us to consider multiple aspects of the executive function from 70 participants. Third, we augment the technical efficiency analysis with a second-stage analysis that controls for differences in terms of age, non-verbal intellectual ability, grammar skill, expressive vocabulary skill, receptive vocabulary skill, SES, and language use. A bootstrap regression is used to mitigate any small sample bias, while an ANCOVA and a k-means nearest-neighbour approach are used as robustness. Fourth, we analyse our bilingual/monolingual dataset using conventional ANCOVA/MANCOVA techniques using the same control variables comparing the results to the technical efficiency analysis.

### Efficiency studies across disciplines

Assessing the performance of organisations such as firms, financial institutions, educational institutions, and hospitals is of interest to investors, regulators, policy-makers, and consumers. Perhaps the simplest performance ratio is in the form of Ouput/Input. The manager of an electricity power plant may get a rough estimate of the performance by assessing output level produced (e.g., electricity in MWh), given a level of input (e.g., barrels of Oil) (Kumbhakar & Tsionas, 2011). This performance ratio combines two important concepts; first that higher values of output are more desirable; second, there is a cost element that needs to be minimised. The owner of a dairy farm may also be interested in benchmarking the performance of his/her firm in a similar manner. The output in this case may be viewed as the milk (in litres) produced by the cows, while the inputs may relate to the number of cows used, the size of the land, the labour quantity and the quality of the feeds (Alvarez & Arias, 2004). With such information, the manager could benchmark the operations against the competition and/or against time and find areas for improvement. For example, Johnes et al. (2014) argue that Islamic banks have lower technical efficiency than commercial banks due to the formers’ business model restrictions that prohibit the issuance of loans to certain types of businesses.

In the above examples, we shall refer to the business entity as a decision-making unit (DMU). The DMU is a flexible definition allowing the generalisation of the technique across a wide range of applications (see Table 1). In general, the DMU may be viewed as a “black-box” entity that transforms inputs into outputs. The term “decision” implies a mental process; in fact, it could be argued that the manager in the above examples would have some control over the production process and/or the output-input mix. However, this does not need to be the case as the DMU could be a jet engine (Bulla et al., 2000).

**Table 1:** Examples of technical efficiency studies

Inputs	DMU	Outputs	References
Example 1	Dairy farms		
Number of cows		Milk (in litres)	Alvarez and Arias (2004)
Size of land (in hectares)			
Labour (in man-equivalent hours)			
Feeds (in tons)			
Example 2	Power plants		
Fuel		Electricity (in MWh)	Kumbhakar and Tsionas (2011)
Labour (in man-equivalent hours)			
Fuel (in tons)			
Capital (in millions USD)			
Example 3	Banks		
Labour (in millions USD)		Loans (in millions USD)	Johnes et al. (2014)
Physical capital (in millions USD)		Securities (in millions USD)	
Financial capital (in millions USD)			
Example 4	Hospitals		
Number of inpatients		Outpatients	Cooper et al. (2007)
Number of doctors			
Number of nurses			
Example 5	Universities		
Total cost		Full-time equivalent UG students	Thanassoulis et al. (2011)
		Full-time equivalent PG students	
		Research income	
		Intellectual property income	
Example 6	Bilingual children		
Non-verbal intellectual ability		Executive function (Accuracy)	This study
Grammar skill		Executive function (RT)	
Expressive vocabulary skill			
Receptive vocabulary skill			
Age			

Up to this point, we have referred to “performance” without giving an appropriate definition. In fact, this is a known issue in certain disciplines as the “true” firm performance is latent, with individual measures (i.e., proxies) not being comprehensive indicators. In the management literature, the creation of competitive advantage of a firm against its competitors is important, as it could enhance a firm’s performance (Douglas & Judge Jr, 2001). On this occasion, *performance* per se would relate to profitability; yet other aspects, such as the firm value may also have been relevant. In the banking literature, capitalisation, profitability, stability, and liquidity could fall under the umbrella term of *performance*; yet multiple indicators exist to separately quantify each of these concepts. Drivers of each of these indicators are not necessarily the same. Ultimately, one may be interested in a holistic *performance* of a bank. Therefore, the challenge lies in combining all the information from a set of indicators to arrive at a meaningful conclusion, which should be generalizable and replicable. Hence, the need for an approach that could capture multiple aspects of the complex organisational structure and present a single, straightforward indicator to the interested parties is apparent.

We assume that each participant is the DMU, with outputs comprising i) paying attention; ii) organisation; iii) maintaining focus; iv) self-monitoring (Diamond, 2013). These skills may be mapped against the three distinct and interrelated processes, namely working memory, inhibition, and switching identified in Miyake et al. (2000). Inputs to the DMU are non-verbal intellectual ability, grammar skill, expressive vocabulary skill, and receptive vocabulary skill.

## Method

### Participants

Our sample comprises 32 bilingual (mean age = 9 years and 1month, *SD* = 2 years and 2 months, 18 females and 14 males) and 38 monolingual (mean age = 9 years and 9 months, *SD* = 1 year and 8 months, 22 females and 16 males) children; a total of 70^6^. The bilingual children are competent in both Greek and English languages to varying degrees. The bilingual children were recruited if at least one of their parents spoke the Greek language with them. The mean age of acquisition is 7 months (*SD* = 1 year and 2 months) for Greek and 2 years and 6 months (*SD* = 2 years and 9 months) for English. We have excluded any trilingual participants.^7^ Children were included in the study if their non-verbal intelligence score was not under 80. In this case, all children had scores over 80. Based on parental and teacher reports, the children did not have any hearing, behavioural, emotional, or mental impairment. More information is included in Table 4 and “Descriptive statistics” section below.

Bilingual Greek–English children were recruited from a Greek supplementary school in the north-west of England. The school offered a Greek-speaking supplementary program for 2.5 to 3.5 hours a week to enhance the reading and writing of the Greek language. This program is supplementary to the mainstream English school that these children attend. Eight of the bilingual children were born in Greece and lived in the UK for more than 2 years at the time of the study. The Greek monolingual control group consisted of children born and based in Greece.

Ethical approval was granted by the College of Arts and Humanities Research Ethics Committee at Bangor University. Information sheets were sent to the head teachers of schools and to the parents and informed consent was obtained before the collection of data. Teachers, parents, and children were provided enough time to express any questions about the nature of the study. Parents and children were informed that they could withdraw at any time, and they were debriefed after the study.

### Materials

#### Parental questionnaire

The children’s language experience was assessed through the Language and Social Background Questionnaire for Children (LSBQ) (Luk & Bialystok, 2013). The LSBQ was forward and backward translated in Greek and it was completed by at least one of the parents/guardians in their preferred language (Greek or English). It consisted of information about the child’s age, grade, date of birth, country of birth, age of onset of all the languages, knowledge of playing a musical instrument, and length of exposure to different educational mediums. The questionnaire also included information about the parents’ language backgrounds. SES was measured as the mean of the highest attained educational level of both parents rated on an eight-point scale. Parental education is the most commonly used index of SES background, is highly predictive of other SES indicators (e.g., income, occupation), and is a better predictor of cognitive performance than other SES indicators (Calvo & Bialystok, 2014). The child’s understanding and speaking in all of their languages was rated on a five-point scale ranging from Poor to Excellent. Language use with parents, siblings, grandparents, neighbours, friends, and caregivers in various situations was measured on a seven-point scale ranging from 1 (only English) to 7 (only Greek/or other language).

#### Non-verbal intelligence

Non-verbal intelligence was assessed using the Kaufman Brief Intelligence Test, Second Edition (KBIT-2) (Kaufman, 2004). It consists of 46 items including a series of abstract images, such as designs and symbols, and visual stimuli, such as pictures of people and objects. Participants were required to understand the relationships among the presented stimuli and complete visual analogies by indicating the relationship between the images by either pointing to the answer or saying its letter. All items include an option of at least five answers thus reducing the chance of guessing. The Matrices non-verbal subtest was individually administered and scored according to the KBIT-2 manual, and percentages for the Matrices scores were obtained for participants.

#### Language measures

##### English language measures

The British Picture Vocabulary Scale, Third Edition - BPVS3 (Dunn & Dunn, 2009) was used to assess the receptive vocabulary of the bilingual and monolingual children in the English language. It is an individually administered, standardised test of Standard English receptive vocabulary for children ranging from 3 years to 16 years and 11 months. In this task, children are asked to select, out of four coloured items in a 2 by 2 matrix, the picture that best corresponds to an English word read out by the researcher. The assessment consists of 14 sets of 12 words of increasing difficulty (e.g., ball, island, fictional). The administration is discontinued when a minimum of eight errors is produced in a single set.

The Clinical Evaluation of Language Fundamentals, Fourth UK Edition - CELF-4UK (Semel et al., 2006) is an individually administered standardised language measure, which is used for the comprehensive assessment of a student’s language skills by combining core subtests with supplementary subtests. The expressive vocabulary subtest was used here to assess the participants’ expressive vocabulary in the English language. This measure is designed for children and adolescents ranging from 5 to 16 years of age. Expressive vocabulary was screened through the Expressive Vocabulary subtest for children. Children were asked to look at a picture and name what they see or what is happening in each picture (e.g., a picture of a girl drawing, the child should give the targeted response ‘colouring’ or ‘drawing’ to score 2 points or the response ‘doing homework’ to score 1 point). The administration is discontinued after seven consecutive zero scores.

The Test for Reception of Grammar, Version 2 - TROG-2 (Bishop, 2003) was used to assess receptive grammar. It is an individually administered standardised test for children and adults and it comprises 80 items of increasing difficulty with four picture choices. Children are asked to select the item that corresponds to the target sentence read out by the researcher. For each grammatical element, there is a block of four target sentences. A block is failed unless all four items of each block are established by the child. The sentences include simple vocabulary of nouns, verbs, and adjectives. If a child fails five consecutive blocks the administration is terminated.

##### Greek language measures

A standard Modern Greek version of the Peabody Picture Vocabulary Task-PPVT (Dunn, 1981) was adapted and used based on the Greek adaptation by Simos et al. (2011). The children clicked on the image, out of four possible choices, that best corresponded to the target word they heard, such as nouns, verbs, or adjectives. There were 173 items of increasing difficulty. If eight incorrect responses were provided to ten consecutive items, then the task was stopped. The answers were scored as correct (1) or incorrect (0).

The Picture Word Finding Test-PWFT (Vogindroukas et al., 2009) is an individually administered standardised measure used to assess standard Modern Greek expressive vocabulary. It is a tool norm-referenced for Greek adapted from the English Word Finding Vocabulary Test - 4th Edition (Renfrew, 1995). The children are presented with 50 black-and-white images consisting of nouns in developmental order. The words included originate from objects, categories of objects, television programs and fairy-tales very familiar to children. A score sheet is used to record the responses provided during testing which are later scored as correct (1) or incorrect (0). The children are asked to name the objects they saw and when they are ready, they move to the following one. The assessment is discontinued after five consecutive wrong replies.

The Developmental Verbal Intelligence Quotient-DVIQ (Stavrakaki & Tsimpli, 2000) was used to assess Greek receptive grammar. It consisted of five subtests used to measure children’s language abilities in expressive vocabulary, understanding metalinguistic concepts, comprehension and production of morphosyntax, and sentence repetition. This was an assessment that measured language development in standard Modern Greek, and it was administered individually. For this study, only the subtest measuring comprehension of morphosyntax was used for both Greek monolingual and Greek–English bilingual children. Each child was given a booklet with 31 pages, each including three images. The researcher read out a sentence and each child was asked to point to the picture that best represented the situation in the sentence. For example, the sentence might have been “μην καπνίζετε” (do not smoke) and the correct answer depicted a “No Smoking” sign. An answer sheet was used to record the child’s answers (as A, B, or C) during testing which were later scored as correct (1) or incorrect (0).

For each of the background language measures, we define percentage scores as the number of correct responses/number of correct and incorrect responses. Bilinguals were assessed on each of these background measures using one test in each language. Percentages were used in order to create a comparable scale for all tests, which allows us to produce a composite measure.

#### Executive function tasks

In this section, we present the administration details for the five executive function tasks that span attention, working memory, inhibition, and shifting. All cognitive tasks were administered on a laptop using the experimental software E-Prime 2.0 (Schneider et al., 2002). E-Prime 2.0 is behavioural experiment software that provides an environment for computerised experiment design and data collection with millisecond precision timing ensuring accuracy of data. We discuss each of these tasks in turn below.

##### Attention task

The Attentional Network Task (ANT) (Fan et al., 2005) was designed to evaluate three different attentional networks: i) alerting; ii) orienting, and iii) executive control (Posner & Petersen, 1990). Participants are asked to indicate the direction (left or right) that the target stimulus (a fish appearing at the centre of the screen) points to. Distance between the participant’s head and the centre of the screen was approximately 50 cm. The child’s task was to press either the right or left key button on the mouse (with the right or left index finger) corresponding to the direction in which the middle fish is swimming. The child was presented with a training block of 16 trials and 128 trials distributed in four experimental blocks. There were breaks in between. During both the training and experimental blocks, auditory feedback was provided to the child.

##### Working memory tasks

The first task was a *Counting recall task*, which was an adaptation of the Automated Working Memory Assessment (Alloway, 2007). The children were presented on the laptop screen a varying number, from four to seven, of red circles and blue triangles on the screen. The children should remember the number of red circles in each image. The images presented begin from one and reach seven. Each experimental block, consisting of one to seven images, consists of four trials. If the child failed to correctly recall three trials in a block, the task stopped.

The second task was a *Backward digit span task* (BDST) and it was adapted from Huizinga et al. (2006). The children began with two training trials in order to understand the task and type the reverse order of the numbers presented. For example, if a child hears the number 7 and 4 they should type 4 and 7. The sequence begins with four trials of two numbers reaching gradually eight numbers. Similarly to the above task, if the child failed to correctly recall three trials in a block the task stopped.

Both tasks were administered in the preferred language of the child. In all cases the preferred language was English for the bilingual children.

##### Inhibition task

The *Nonverbal Stroop task* was adapted from Lukács et al. (2016) and consisted of stimuli of arrows pointing upwards, downwards, left and right. Three experimental blocks of 60 trials each were presented to the children. The aim was to select the direction that the arrows indicated regardless of their position on the screen. The children used the arrow buttons on the laptop’s keyboard. The first was the control block and arrows were presented in the middle of the screen (Stroop base). In the second block, which was the congruent block, the direction of the arrows matched their position on the screen (e.g., an arrow indicating upwards was presented at the top of the screen) (Stroop congruent). Finally, the third experimental block was the incongruent block. Here the direction of the arrows was the opposite compared to their position on the screen (e.g., an arrow indicating upwards was presented at the bottom of the screen) (Stroop incongruent). During the administration of the task, the second and the third blocks are randomly mixed to enhance the conflict effect.

For accuracy measures, the number of correct answers for the incongruent items was subtracted from the number of correct answers for the congruent items. The difference in reaction times for congruent and incongruent trials represents the inhibition cost.

##### Shifting task

All children were also administered one shifting task, the *Colour-shape task.* This task included three blocks each, where children were presented with two shapes (triangle, circle) coloured either red or blue. The same buttons, one for the left hand and on for the right, corresponded to one of the choices (circle–triangle, red–blue). In the first two experimental blocks, the children’s task was to recognise the shape of the stimulus and ignore their colour or the reverse. The stimuli were presented in the top half and bottom half of the screen, respectively. In the third block, they were required to alternate between colour and shape depending on their location on the screen. Cues directing the participant to the relevant dimension are presented simultaneously with the stimuli on all trials, in all blocks. The first two blocks contained 32 trials each, while the third block contained 64. The number of shifting and non-shifting sequences within the third block was balanced. The difference in reaction times for the first two (non-shifting) and the third (shifting) block represents the shifting cost.

### Procedures

A pilot study with four children was conducted before the actual data collection. As a result of the pilot study, the choice of the above fixed order of tasks was such so the children did not feel tired or uninterested. After the end of each session, the researcher thanked the child for their participation. All children participated enthusiastically.

The children were tested individually in a quiet school classroom setting, during one session in Greek and one session in English that lasted 40 min on average. The second session was conducted no more than 1 month’s time after the first one. The researcher informed the children that they would play some games. Parents were administered the questionnaire (LSBQ) and returned it to the researcher, or the classroom teacher, or the school’s head teacher.

The first session was the Greek session for the bilingual participants. Each child completed the tasks in the following fixed order: i) Greek adapted PPVT, ii) ANT, iii) PWFT, iv) Colour shape task, v) Nonverbal Stroop task, and vi) DVIQ. The second session was the English session for the bilingual participants. Each child completed the tasks in the following fixed order: i) KBIT-2, ii) BDST, iii) BPVS, iv) counting recall task, v) CELF-4, and vi) TROG-2.

The Greek monolingual children completed the tasks in the following fixed order: i) Greek adapted PPVT, ii) ANT, iii) PWFT, iv) Colour shape task, v) Nonverbal Stroop task, vi) DVIQ, vi) KBIT-2, vii) BDST, viii) Counting recall task.

### Technical efficiency

In this section, we introduce the concept of technical efficiency, which may be viewed as a special case of a performance ratio. We use a random sample from our dataset and assume that each participant is a decision-making unit (DMU) that produces two outputs from two inputs. The outputs are the accuracy scores on two executive function tasks; the BDST and the Counting recall. The inputs are a measure of the non-verbal intellectual ability (KBIT-2) and a measure of the grammar skill (DVIQ). Ultimately, we are interested in comparing the performance of the DMUs. We illustrate three cases; case A considers one Output and one Input; case B uses two Outputs and one Input; case C uses two Outputs and two Inputs.

Table 2, Panel A, presents the output and input values for each of the ten participants of the random sample. Panel B calculates an array of performance measures associated with each of the three cases outlined above.

**Table 2:** Performance ratios and technical efficiency

Participant	A	B	C	D	E	F	G	H	I	J
Panel A: Random sample details										
KBIT-2	89.13	89.13	82.61	54.35	69.57	52.17	60.87	67.39	71.74	82.61
DVIQ	96.77	96.77	87.10	93.55	90.32	90.32	58.06	38.71	96.77	58.06
BDST	75.00	75.00	75.00	66.67	60.00	66.67	66.67	66.67	75.00	68.42
Count recall	81.63	81.63	60.71	57.14	75.00	50.00	35.71	46.43	57.14	71.43
Panel B: Performance ratios										
Case A: 1 Output / 1 Input										
BDST / KBIT-2	0.841	0.841	0.908	1.227	0.863	1.278	1.095	0.989	1.045	0.828
Case B: 2 Outputs / 1 Input										
BDST / KBIT-2	0.841	0.841	0.908	1.227	0.863	1.278	1.095	0.989	1.045	0.828
Count recall /KBIT-2	0.916	0.916	0.735	1.051	1.078	0.958	0.587	0.689	0.797	0.865
Case C: 2 Outputs / 2 Inputs										
BDST / KBIT-2	0.841	0.841	0.908	1.227	0.863	1.278	1.095	0.989	1.045	0.828
Count recall / KBIT-2	0.916	0.916	0.735	1.051	1.078	0.958	0.587	0.689	0.797	0.865
BDST / DVIQ	0.775	0.775	0.861	0.713	0.664	0.738	1.148	1.722	0.775	1.178
Count recall / DVIQ	0.844	0.844	0.697	0.611	0.830	0.554	0.615	1.199	0.590	1.230
Technical efficiency	0.793	0.793	0.752	0.789	0.796	0.772	0.809	1.000	0.738	0.934

*Notes.* The table reports inputs and outputs used in the efficiency analysis for a random sample of ten participants from our datasets. The outputs are the accuracy scores (on a percentage scale) on BDST and Count recall executive function tasks. The outputs are the accuracy scores of two executive function tasks of working memory; BDST and Counting recall. The inputs are measures of non-verbal intellectual ability (KBIT-2) and grammar skill (DVIQ). Performance ratios are presented in panel B for the cases of 1 output / 1 input (Case A), 2 outputs / 1 input (Case B) and 2 outputs / 2 inputs (Case C). The technical efficiency row presents the equivalent measure, which is estimated using data envelopment analysis (DEA) as described in “Descriptive statistics” section

**Fig. 1:**
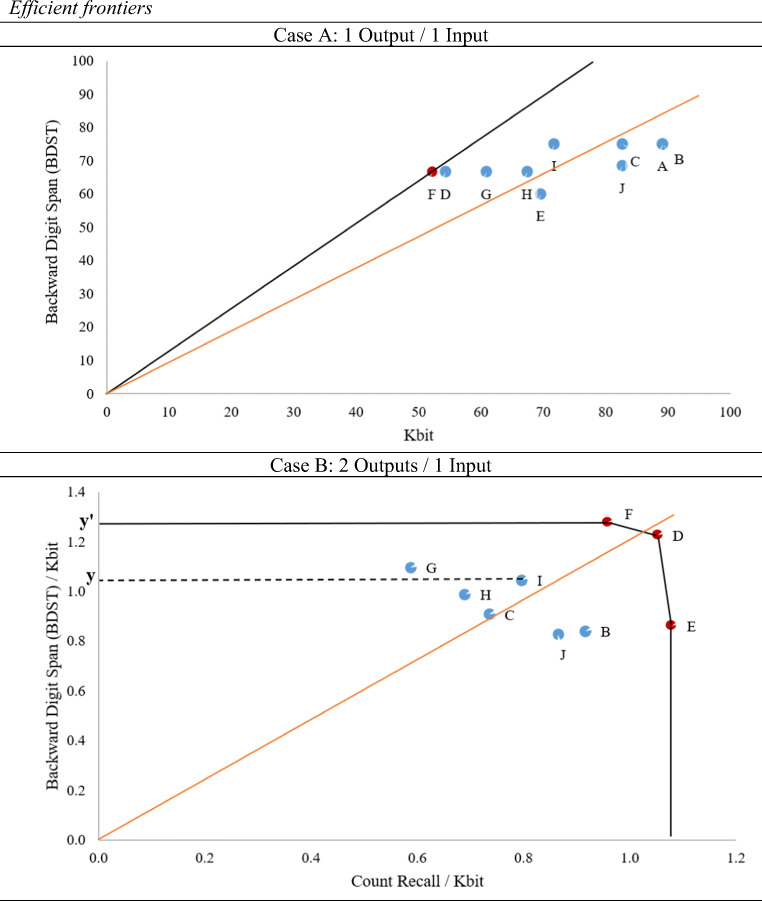
Efficient frontiers. *Notes.* The figure shows the efficient frontier (*solid black line*) in the case of 1 output / 1 input (Case A), and 2 outputs / 1 input (Case B). The *orange line* represents the best-fit line from a regression model. The outputs are the accuracy scores in two executive function scores, BDST (Case A and B) and Count recall (Case B). The input is the non-verbal intellectual ability as proxied by the KBIT-2 score (Case A and B). The ten participants labelled A-J are a random sample from our dataset

In case A, the ratio BDST / KBIT-2 may be viewed as a performance measure where higher values denote a participant with a superior performance; i.e., a higher accuracy score in the BDST measure, using a lower KBIT-2 score. Participant F has the highest value (1.278), hence may be viewed as the one with the best performance, or the most efficient. That is, s/he is producing the highest BDST accuracy score by using the lowest KBIT-2 score. A graphical representation of the ten participants is given in Fig. 1a. The line that connects the axis origin (black line) to point D (the left-most in the graph) is the efficient frontier and envelops all the other points. By contrast, a regression line (orange line) goes through the middle of these points; a direct consequence of the estimation technique used. As such, while the regression line considers the “average” as the benchmark unit, by allowing some to over-perform and others to under-perform, the frontier analysts consider the efficient (i.e., best-practice) unit as the benchmark; thus letting all others to under-perform.

In case B, the ratios BDST / KBIT-2 and Counting recall / KBIT-2 are defined. Points F, D, and E are of special attention as they are the furthest away from the axis origin, hence they represent the best-performers (i.e., efficient ones). The participants represented by these three points represent efficient combinations in the sense that they produce the maximum output for a given level of input. Contrary to case A, the efficient frontier here is a piecewise linear frontier that is made up of the efficient DMUs and envelops all the inefficient combinations. For example, point *I* lies inside the frontier and has an efficiency score of Oy/Oy’, which means that there is a margin of improvement in the performance of participant *I* by Oy’-Oy (i.e., the distance between point *I* and the efficient frontier).

Case C would require the ratios BDST / KBIT-2, Counting recall / KBIT-2, BDST / KBIT-2 and Counting recall / DVIQ to be computed. However, in this case visual representation would have to be multidimensional. A particular challenge that was made apparent in case B is that the points (F, D, E) are all efficient but have a different output/input mix. For example, point F is superior in terms of BDST, while point E in terms of Counting recall. The fact that the output/input mix would vary among DMUs becomes more apparent as outputs and inputs considered increase. Consequently, it is difficult to identify the participant with the overall best performance, unless we assign some “desirability” on the outputs (and similarly the inputs). For example, this could take the form of a higher accuracy in the BDST having a higher value than in the Counting recall.

To address the issue, Charnes et al. (1978) introduced the concept of technical efficiency in the form of a linear optimisation model – the CCR model. The novelty lies in the use of weighted outputs and weighted inputs to form a performance measure, known as *technical efficiency*. Technical efficiency may be viewed as a ratio where, on the nominator (denominator) each output (input) is assigned a weight. The weight, which lies between 0 and 1, is universal for all the DMUs, and could be viewed as a measure of the relative desirability of the outputs and inputs.

A linear optimisation technique that maximises the overall technical efficiency of the system is used to estimate the weights (Charnes et al., 1978). Hence, the weights, and consequently any ranking of outputs and inputs that is implied, is determined from the data themselves without any a priori information or assumptions.

Mathematically, and starting from the case of two outputs and two inputs (i.e., Case C), the technical efficiency ratio for a single DMU is given as:

1 $$ TE=\frac{u_1{y}_1+{u}_2{y}_2}{v_1{x}_1+{v}_2{x}_2} $$

where *y*_1_ and *y*_2_ being the BDST and Counting recall accuracy scores (Outputs); *x*_1_ and *x*_2_ being the KBIT-2 and DVIQ scores (inputs); *u*_1_, *u*_2_, *v*_1_ and *v*_2_ are output and input weights, respectively.

We can generalise this to the case of *R* outputs and *M* inputs as follows:

2 $$ {TE}_j=\frac{{\overset{\sim }{u}}_1{y}_{1,j}+{\overset{\sim }{u}}_2{y}_{2,j}+\dots +{\overset{\sim }{u}}_R{y}_{R,j}}{{\overset{\sim }{v}}_1{x}_{1,j}+{\overset{\sim }{v}}_2{x}_{2,j}+\dots +{\overset{\sim }{v}}_M{x}_{M,j}} $$

Here we also add the subscript *j* which denotes the DMU with *j* = 1, 2, …, *N* as well as the tilde on top of the weights to denote that these are estimated through linear optimisation. Note that as the weights are common across all DMUs, they do not carry the *j* subscript.

The linear optimisation works by maximising the sum of *TE*_*j*_ across all DMUs subject to the *TE*_*j*_ being bounded between 0 and 1 (where 1 is assigned to the efficient DMUs) for each DMU, and to the weights being non-negative.^8^ Mathematically:

3 $$ \underset{u,v}{\max}\sum \limits_{j=1}^N{TE}_j $$

4 $$ \mathrm{subject}\ \mathrm{to}:\left\{\begin{array}{c}0\le {TE}_j\le 1\\ {}{\overset{\sim }{u}}_1,{\overset{\sim }{u}}_2,\dots, {\overset{\sim }{u}}_R\ge 0\\ {}{\overset{\sim }{v}}_1,{v}_2,\dots, {\overset{\sim }{v}}_R\ge 0\end{array}\right. $$

### Data transformations

In our case, each child produces certain outputs while receiving certain inputs. We consider the output to be the executive function score, which may be viewed as a proxy for brain performance.

As per the Miyake et al. (2000) classification, three distinct and interrelated components of executive function are defined. These relate to an individual’s ability to switch between various tasks (switching/shifting), the ability to maintain and process information in mind (working memory), and the ability to suppress irrelevant information at any given moment (inhibition). Performance in each of these categories is assessed via the following tasks: i) BDST, ii) Counting recall, iii) Colour shape, iv) Non-verbal Stroop (Stroop), v) ANT. All of these tasks and their administration procedure have been explained in an earlier section.

In each task we record: i) the accuracy (ACC); ii) the response time (RT) of the child, which form our two outputs. The accuracy for each task and each child is calculated as the average accuracy over the respective number of trials that each task consists of, and ranges theoretically between 0 and 1. For tasks that have congruent and incongruent trials, we use the average accuracy. Empirically, extreme points are not observed, thereby the tasks are appropriate for the children’s age. The higher the accuracy the better the performance of the child.

The response time is measured in milliseconds and is only considered for the correct answers to test questions. The lower the response time, the faster the response is given. Consistent with the literature, we exclude any response time that is below 200 ms (Antoniou et al., 2016). We also carry out an outlier treatment in line with Purić et al. (2017), where we trim response times that lie outside of a 3 standard deviations bound.^9^ As the two output variables are inversely coded, we consider the inverse of response time and dub this as *response speed* (1/RT).^10^ Hence, the two outputs in our case are: i) accuracy (*y*_1_); ii) response speed (*y*_2_). The inputs are as follows: i) non-verbal intellectual ability (*x*_1_); ii) grammar skill (*x*_2_); iii) expressive vocabulary skill (*x*_3_); iv) receptive vocabulary skill (*x*_4_); v) age (*x*_5_).

The grammar, expressive vocabulary, and receptive vocabulary skills of monolingual children are assessed in Greek using the DVIQ, the PWFT, and the Greek receptive vocabulary test, respectively. The grammar, expressive vocabulary, and receptive vocabulary skills of bilingual participants are assessed in Greek using the same measures as with the monolinguals and in English using the equivalent English tests, namely TROG-2, CELF-4 and BPVS, respectively. With regards to the intellectual ability, we used the Matrices subtest, which is the non-verbal component of the KBIT-2. Table 3 presents information about the mapping of the tasks for each group of participants.

**Table 3:** Task mapping per group

Measures	Administered task	Category	Bilinguals	Greek monolinguals
Accuracy (*y*_1_) Response speed (*y*_2_)	BDST	Working memory	✓	✓
	Counting recall	Working memory	✓	✓
	Colour shape	Shifting	✓	✓
	Non-verbal Stroop	Inhibition	✓	✓
	ANT	Inhibition/Attention	✓	✓
Non-verbal intellectual capacity (*x*_1_)	KBIT-2		✓	✓
Grammar skill (*x*_2_)	DVIQ		✓	✓
	TROG-2		✓	—
Expressive vocabulary skill (*x*_3_)	BWFT		—	✓
	CELF-4		—	—
Receptive vocabulary skill (*x*_4_)	Greek receptive vocabulary		—	✓
	BPVS		—	—

*Notes.* The table presents the outputs and inputs of the technical efficiency analysis, with information on the mapping of the tests in each group

To arrive at comparable estimates of grammar, expressive vocabulary, and receptive vocabulary skills, we standardise the scores of the monolinguals and bilinguals. As the bilinguals have two measures for each skill, one in Greek and another in English, we follow three strategies to arrive at a composite measure of the respective skill. In the most naïve and easiest-to-implement strategy, we assume that all bilinguals are balanced between English and Greek, hence their composite score would be a simple weighted average of the respective tasks, and this represents Composite Score 1 (CS1). As the balanced bilingual assumption may be strong, we introduce a second, more realistic composite score (CS2) that assumes that bilinguals may be more competent in a particular language. Hence, under CS2, the composite measure is a weighted average of the individual tasks, with the weights calculated from the relative performance of the participants in the Greek and English versions of the test. Composite Score 3 (CS3) is similar to CS2 with the only difference being that the relative weights are derived from the parental questionnaire; hence the relative competency level is self-declared. In the following analysis, we present the results based on CS2, and we compare with the results of CS1 in the robustness section.^11^

Similar to regression models, a DEA analysis needs to be “well specified” in the sense that relevant variables should be included in the specification. In case of regression, a minimum number of observations is required for estimation; statistical inference (e.g., hypothesis testing) requires additional observations and/or bootstrap techniques for small samples. Due to the DEA’s non-parametric nature, minimum sample size has no formal statistical basis. However, DEA’s discriminatory power depends on the relative numbers of inputs, outputs, and DMUs in the sample. As a rule of thumb, the number of DMUs should be at least 2–3 times higher than the inputs and outputs combined (Banker et al., 1989; Golany & Roll, 1989). In our case, the number of DMUs is at least seven times higher than the combined inputs and outputs.

### Second-stage analysis

The technical efficiency estimate from the previous step may be used as the dependent variable in subsequent analysis. We investigate differences in the technical efficiency of monolingual and bilingual children in a second-stage analysis. We use three estimation methods: i) an ANCOVA, which is widely used in the literature; ii) a regression with bootstrap corrected standard errors that corrects for potential small sample bias (Cameron & Trivendi, 2005); and iii) a k-means nearest-neighbour matching technique. We opt for the k-means nearest neighbour as it is a non-linear, non-parametric technique that matches observations with similar characteristics. The advantage of k-means nearest-neighbour matching is that it does not rely on a formal model (like propensity score does); thus, being more flexible. Like the propensity score approach, it can match observations on both categorical and continuous variables. However, when matching on continuous variables, a bias-corrected nearest-neighbour matching estimator is necessary (Abadie & Imbens, 2006, 2011). More information is provided in Technical Appendix 2.

We allow for three formulations in each estimation method, hereafter referred to as Specifications A to C, respectively. These specifications are progressively less restrictive as they allow for decreasing similarities between the participants. In particular, specification A controls for differences with respect to non-verbal intellectual ability, grammar skill, expressive vocabulary skill, receptive vocabulary skill and age. Specification B further adds SES to specification A, while specification C further adds language use to specification B.

## Results

### Descriptive statistics

Table 4 and presents key descriptive statistics for the variables utilised in the analysis. The mean, standard deviation, and median for the bilinguals and monolinguals is reported alongside an ANOVA between-group test. Lack of statistical significance in the F-statistic suggests no group differences between the bilinguals and monolinguals.^12^

**Table 4:** Descriptive statistics

Measure	Task	Units	Bilinguals (*n* = 32)			Greek monolinguals (*n* = 38)			F-stat	η^2^
			Mean	*SD*	Median	Mean	*SD*	Median		
Backward digit span (ACC)		%	0.624	0.097	0.628	0.668	0.080	0.667	4.47^**^	0.062
Counting recall (ACC)		%	0.494	0.144	0.464	0.478	0.139	0.500	0.22	0.003
Colour shape (ACC)		%	0.821	0.083	0.836	0.830	0.086	0.848	0.18	0.003
Stroop (ACC)		%	0.894	0.117	0.933	0.900	0.109	0.947	0.06	0.001
ANT (ACC)		%	0.931	0.089	0.965	0.954	0.067	0.992	1.56	0.022
Backward digit span (RT)		ms	933.30	289.95	956.08	807.11	271.59	792.27	3.53	0.049
Counting recall (RT)		ms	2394.90	1215.61	2149.13	1887.87	1179.38	1523.10	3.12	0.044
Colour shape (RT)		ms	921.67	186.50	923.48	962.89	147.44	984.83	1.07	0.015
Stroop (RT)		ms	861.46	298.93	792.22	784.92	195.46	741.64	1.65	0.024
ANT (RT)		ms	912.30	255.63	865.50	861.81	189.42	866.88	0.90	0.013
Non-verbal intellectual ability	KBIT-2	%	61.75	15.35	64.13	60.93	14.35	58.70	0.05	0.001
Grammar skill	DVIQ	%	82.96	15.05	87.10	92.19	6.12	93.55	11.96^***^	0.150
Expressive vocabulary skill	PWFT	%	45.56	25.97	52.00	83.05	8.28	84.00	70.84^***^	0.510
Receptive vocabulary skill	Greek receptive vocabulary	%	44.20	20.74	46.24	70.46	11.75	71.68	44.16^***^	0.394
Grammar skill	TROG-2	%	71.72	18.78	72.50	—	—	—	—	—
Expressive skill	CELF-4	%	57.86	20.50	56.48	—	—	—	—	—
Receptive vocabulary skill	BPVS	%	63.73	15.16	61.90	—	—	—	—	—
Age		Years	9.14	2.24	8.90	9.77	1.69	9.91	1.81	0.026
SES		%	76.56	19.31	75.00	58.55	12.79	56.25	21.76^***^	0.242
Greek proficiency		%	79.69	23.07	90.00	96.32	7.86	100.00	17.39^***^	0.204
English proficiency		%	92.50	10.78	100.00	48.68	28.30	55.00	68.24^***^	0.501
Other proficiency		%	14.38	24.62	0.00	12.89	25.56	0.00	0.06	0.001
Greek language use		%	49.90	22.73	51.14	94.77	7.88	96.51	129.80^***^	0.656
Music		Binary	0.50	0.51	0.50	0.32	0.47	0.00	2.47	0.035
Years in Greek school		Years	0.47	1.11	0.00	5.00	1.80	5.00	153.54^***^	0.693
Years in supplementary school		Years	3.43	3.47	2.57	2.75	0.00	0.00	69.55^***^	0.506
Total Greek education		Years	3.94	2.39	4.00	5.00	1.80	5.00	4.49^**^	0.062

*Notes.* The table shows descriptive statistics for the executive function tasks (accuracy and response times) and other variables of the dataset. n denotes the observations, *SD* denotes the standard deviation. F-stat and η^2^ correspond to the between-subjects ANOVA tests. ***, **, * denote statistical significance at the 1, 5, and 10% significance level, respectively

**Fig. 2:**
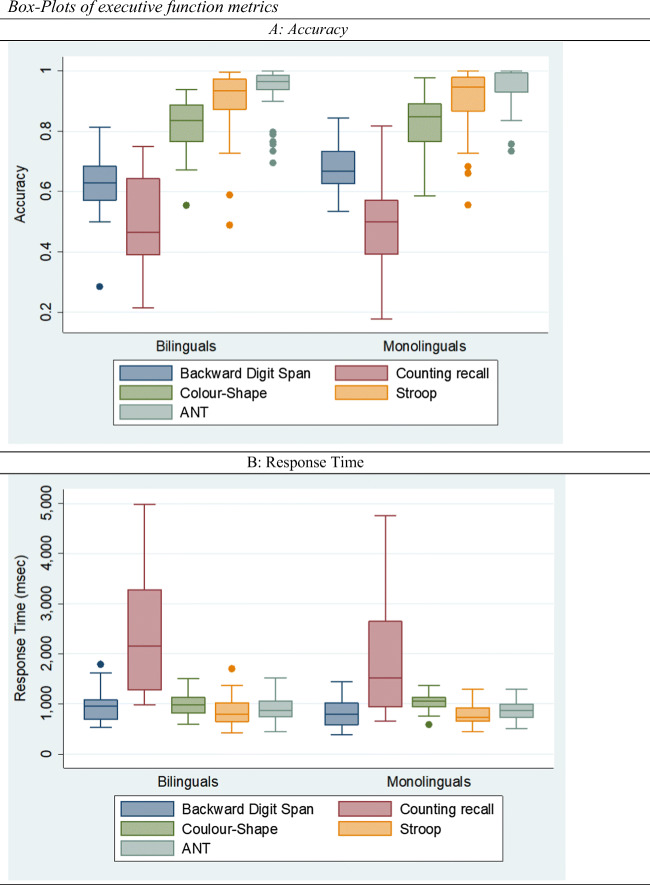
Box-plots of executive function metrics. *Notes.* The figure reports box plots of accuracy and response time metrics for bilinguals and monolingual groups

**Fig. 3:**
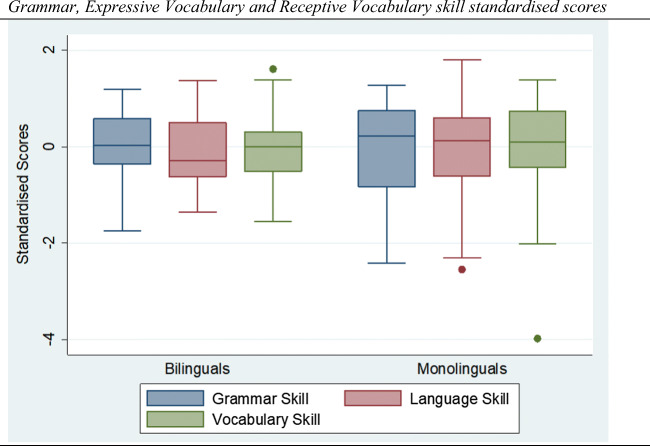
Grammar, expressive vocabulary, and receptive vocabulary skill standardised scores. *Notes.* Standardised grammar, expressive vocabulary, and receptive vocabulary skill metrics for bilinguals and monolingual groups

A first inspection of the executive function accuracy and response times scores (also see Fig. 2) does not suggest any between-group differences, with the exception of the accuracy score in the BDST task (*F*(1, 68) = 4.47, *p* < .05). A comparison of non-verbal intellectual ability and age between the two groups does not suggest any significant difference (*F*(1, 68) = 0.05, ns) and (*F*(1, 68) = 1.81, *p* > .10). A comparison of the Greek versions of the grammar (*F*(1, 68) = 11.96, *p* < .001), vocabulary (*F*(1, 68) = 44.16, *p* < .001) and language (*F*(1, 68) = 70.84, *p* < .001) tasks suggests significant between-group differences (also see Fig. 3), which is consistent with the findings of Bialystok and Craik (2010).

The bilinguals have significantly higher SES compared to the monolingual peers (*F*(1, 68) = 21.76, *p* < .001), higher English proficiency score (*F*(1, 68) = 68.24, *p* < .001) and lower Greek proficiency score (*F*(1, 68) = 17.39, *p* < .001). Proficiency in other languages is comparable in both groups (*F*(1, 68) = 0.20, ns).^13^ The two groups show a significant difference in terms of Greek language use, with the monolinguals using the Greek language significantly more (*F*(1, 68) = 129.80, *p* < .001) compared to the bilinguals, as perhaps expected. The proportion of participants that play a musical instrument is comparable between the two groups (*F*(1, 68) = 2.47, *p* > .10).^14^ Years in Greek school is significantly higher for the monolinguals (*F*(1, 68) = 153.54, *p* < .001) as they have always been studying in a Greek school in Greece. The majority of the bilingual cohort (25 out of 32 participants) never attended a Greek school in Greece, while the remaining seven attended one for a period of 1–4 years.^15^ Instead, the bilinguals attended a supplementary (Greek) school in England, which is additional to their formal English education.^16^ So the overall exposure to Greek education is comparable between the two groups. The variable Total Greek Education shows the total exposure of a participant to the Greek educational system, whether formal in Greece, or informal (i.e., supplementary) in the UK. A between-groups test reveals only mild difference (*F*(1, 68) = 4.49, *p* < .05) in favour of the Greek monolinguals.

The correlations between the accuracy scores, response times as well as age, SES, non-verbal intellectual ability, grammar, expressive vocabulary, and receptive vocabulary score are reported in Appendix Table 11. Positive and significant coefficients between all the accuracy scores of the tasks are evidenced. This suggests a similarity in the performance of the participants across the tasks. The fact that inhibition tasks are positively correlated is in line with the Paap and Greenberg (2013) suggestions. In particular, we find significant correlation between Stroop and Colour shape tasks in terms of accuracy scores (*r* = 0.45, *p* < .01) and of response times (*r* = 0.57, *p* < .01). However, we also document significant and positive correlations between working memory and inhibition tasks. For example, BDST and Stroop (*r* = 0.57, *p* < .01) that provides empirical support to the fact that the underlying cognitive processes may be interrelated or that the proxies used may not tap solely on these processes (task impurity problem). Negative and significant correlations between accuracy and RT scores as perhaps expected, for example within the Stroop task we observe a negative and significant correlation (*r* = – 0.41, *p* < .01). All other variables have the expected relationship with accuracy and RT scores of the tasks, with the exception of SES that does not exhibit any significant relationship. For example, higher IQ is positively correlated with accuracy scores and negatively correlated with RTs.^17^

### Efficiency estimates

Table 5 presents the technical efficiency estimates of bilinguals and monolinguals. Under panel A, we report technical efficiency estimates when using each executive function task’s accuracy and response speed as outputs. In panel B, we combine the information from multiple executive function tasks in two variants, namely the “accuracy” and the “response speed”. The former uses the accuracy scores of all five tasks, while the latter uses their respective response speed. The “All” variant includes both the accuracy and the response speed from all five executive function tasks. The choice of inputs is always the same, which are the non-verbal intellectual ability, grammar skill, expressive vocabulary skill, receptive vocabulary skill and age.^18^ A battery of statistical tests is performed for the between-group differences. The estimated Cronbach’s alpha (*a* = 0.93) indicates strong reliability of the technical efficiency variables.

**Table 5:** Technical efficiency estimates by group

	Monolinguals (*n* = 38)			Bilinguals (*n* = 32)			Mean % gain	Anova F-test	T-test	MW-test	KS-test
Executive function task	Mean	*SD*	Median	Mean	*SD*	Median					
Panel A											
Backward digit span	0.756	0.107	0.738	0.837	0.137	0.844	10.25	7.78^***^	2.73^***^	2.96^***^	0.41^***^
Counting recall	0.645	0.092	0.623	0.755	0.145	0.732	15.75	14.78^***^	3.70^***^	3.60^***^	0.50^***^
Colour shape	0.649	0.144	0.647	0.794	0.152	0.771	20.19	16.78^***^	4.08^***^	3.59^***^	0.40^***^
Non-verbal Stroop	0.642	0.126	0.617	0.760	0.151	0.721	16.86	12.78^***^	3.52^***^	3.67^***^	0.48^***^
ANT	0.622	0.111	0.601	0.755	0.157	0.744	19.36	17.12^***^	4.02^***^	3.83^***^	0.53^***^
Total	0.663	0.116	0.645	0.780	0.148	0.763	16.32				
Panel B											
Accuracy	0.793	0.101	0.769	0.897	0.093	0.908	12.41	20.12^***^	4.52^***^	4.08^***^	0.46^***^
Response speed	0.588	0.157	0.557	0.717	0.212	0.655	19.77	8.50^***^	2.84^***^	2.87^***^	0.37^***^
All	0.796	0.099	0.775	0.905	0.094	0.908	12.85	21.94^***^	4.70^***^	4.34^***^	0.49^***^

*Notes.* The table presents DEA technical efficiency estimates for the monolingual and bilingual groups of children of our sample. The outputs in each executive function task are: i) accuracy, and ii) response speed. The outputs of all five executive function tasks are utilised in the “All” variant. The “accuracy” and “response speed” variants use the accuracy scores and response speed scores of all executive function tasks, respectively. Five inputs are utilised, namely: i) non-verbal intellectual ability, ii) grammar skill, iii) receptive vocabulary skill, iv) expressive vocabulary skill, v) age. The weighting scheme for the bilingual inputs is based on Composite score 2 (see “Second stage analysis” section). For each group, we present the mean, standard deviation, and median of technical efficiency, the logarithmic percentage gain where a positive value indicates that bilinguals are more efficient than monolinguals. A battery of tests is presented including an ANOVA F-test and a bootstrap *t* test for the equality of means between the two groups, a Mann–Whitney (MW) test for the equality of medians between the two groups, a Kolmogorov–Smirnov (KS) test for the equality of the distribution of efficiency scores in the two groups. ^***^, ^**^, ^*^ denote statistical significance at the 1, 5, and 10% significance levels, respectively

A cursory inspection of panel A results suggests that bilinguals exhibit higher technical efficiency by 16.32% on average compared to their monolingual counterparts. Depending on the executive function task, the gain ranges between 15.75% (Counting recall) (*F*(1, 68) = 14.78, *p* < .001) and 20.19% (Colour shape) (*F*(1, 68) = 16.78, *p* < .001). For example, the average technical efficiency of bilinguals for the BDST executive function task is at 0.836 against the 0.756 of the monolingual cohort. This suggests that the bilinguals are about 10.25% better at utilising their available inputs than the monolinguals. Panel B results corroborate our previous findings, with bilinguals being around 12.85% more efficient than the monolinguals based on the “All” variant and the effect is significant (*F*(1, 68) = 21.94, *p* < .001). An investigation of the “accuracy” and “response speed” variants suggests that the higher efficiency scores of the bilinguals are mainly driven by their relatively faster responses compared to the monolingual group.

### Second-stage analysis

Table 6 presents the results of the second-stage analyses. Panel A controls for age, non-verbal intellectual ability, grammar skill, expressive vocabulary skill, and receptive vocabulary skill. Panel B further controls for SES. Panel C further controls for language use. The “Margin” column reports the estimated marginal effect of the between-group differences, where a positive value indicates that the bilinguals exhibit superior technical efficiency compared to their monolingual peers.^19^ The main finding is that after controlling for an extended array of controls, the superior technical efficiency of bilinguals found in “Comparison to an alternative dataset - The Antoniou et al. (2016) dataset” section persist.

**Table 6:** Second-stage analysis

Executive function test	I (ANCOVA)			II (Bootstrap regression)				III (k-means NN)		
	Margin	F-stat	η^2^	Margin	SE	t-stat	Adjusted R^2^	Margin	SE	t-stat
Panel A: Specification A										
Backward digit span	0.056^**^	4.750	0.070	0.056^**^	0.026	2.120	0.348	0.071^**^	0.030	2.390
Counting recall	0.085^***^	20.750	0.247	0.085^***^	0.020	4.260	0.671	0.087^***^	0.020	4.350
Colour shape	0.147^***^	16.700	0.210	0.147^***^	0.036	4.130	0.215	0.161^***^	0.045	3.580
Non-verbal Stroop	0.093^***^	16.190	0.204	0.093^***^	0.024	3.920	0.610	0.090^***^	0.023	3.960
ANT	0.105^***^	21.800	0.257	0.105^***^	0.024	4.400	0.631	0.107^***^	0.025	4.240
Accuracy	0.084^***^	18.360	0.225	0.084^***^	0.020	4.070	0.486	0.088^***^	0.023	3.790
Response speed	0.089^***^	11.010	0.149	0.089^***^	0.028	3.180	0.687	0.092^***^	0.030	3.050
All	0.088^***^	21.610	0.255	0.088^***^	0.020	4.400	0.529	0.092^***^	0.022	4.120
Panel B: Specification B										
Backward digit span	0.033	1.370	0.022	0.033	0.030	1.100	0.366	0.073^**^	0.035	2.080
Counting recall	0.053^***^	7.490	0.107	0.053^**^	0.021	2.510	0.720	0.076^**^	0.037	2.040
Colour shape	0.153^***^	13.810	0.182	0.153^***^	0.039	3.910	0.203	0.182^***^	0.049	3.710
Non-verbal Stroop	0.067^**^	6.890	0.099	0.066^**^	0.025	2.710	0.633	0.088^**^	0.042	2.130
ANT	0.082^***^	10.720	0.147	0.081^***^	0.025	3.240	0.647	0.098^**^	0.049	2.020
Accuracy	0.074^***^	11.150	0.152	0.074^***^	0.024	3.050	0.485	0.092^***^	0.028	3.210
Response speed	0.078^**^	6.510	0.095	0.078^**^	0.032	2.470	0.685	0.127^**^	0.056	2.280
All	0.080^***^	14.010	0.184	0.081^***^	0.023	3.470	0.525	0.093^***^	0.028	3.290
Panel C: Specification C										
Backward digit span	0.043	0.870	0.014	0.043	0.046	0.920	0.398	0.024	0.076	0.310
Counting recall	0.011	0.140	0.002	0.011	0.032	0.370	0.727	0.036	0.047	0.780
Colour shape	0.179^***^	6.890	0.101	0.179^***^	0.068	2.650	0.193	0.177^*^	0.107	1.660
Non-verbal Stroop	0.036	0.077	0.013	0.036	0.041	0.089	0.632	0.076	0.056	1.360
ANT	0.010	0.060	0.001	0.010	0.039	0.250	0.670	0.329	0.065	0.510
Accuracy	0.047^*^	1.660	0.026	0.047	0.038	1.220	0.484	0.103^*^	0.053	1.950
Response speed	0.038	0.580	0.009	0.038	0.051	0.760	0.686	0.035	0.054	0.640
All	0.054^**^	2.320	0.036	0.054^*^	0.037	1.440	0.525	0.103^**^	0.052	1.970

*Notes.* The table presents second-stage results for the technical efficiency estimated in a previous section. The technical efficiency is the dependent variable from the previous step and three estimation techniques are used; i) ANCOVA, ii) Regression with bootstrapped standard errors, iii) k-means nearest neighbours. The margin columns present the estimated coefficient (i.e., marginal effect) of the bilingual binary variable, which takes the value 1 for bilinguals, 0 otherwise. Panel A controls for age, non-verbal intellectual ability, grammar skill, expressive vocabulary skill, and receptive vocabulary skill. Panel B further controls for SES. Panel C further controls for language use. ***, **, * denote statistical significance at the 1, 5, and 10% significance level, respectively

The results of the “All” variants are particularly interesting as these combine the information from all five executive function tasks. The marginal effect across all estimation methods and specifications is positive and statistically significant (${\hat{\beta}}_{II}=0.054,p<.10$). This suggests that the bilinguals exhibit, on average, between 5.4% and 10.3% superior technical efficiency compared to their monolingual peers.

A comparison of panel A and panel C finds the former with more statistically significant coefficients. However, once we add all the covariates certain marginal effects drop from statistical significance at conventional levels. This is particularly the case for the individual executive function tasks. In particular, technical efficiency based on the BDST executive function task is statistically significant at Panel A $\left({\hat{\beta}}_{II}=0.056,p<.05\right)$ but not at panel C $\left({\hat{\beta}}_{II}=0.043,\mathrm{ns}\right)$, which highlights the importance of SES and language use in isolating the bilingual effect. This is in line with the comments in Paap (2018) about improper controlling of factors may reveal a bilingualism advantage.

## Robustness tests

### Comparison with conventional designs

In this section, we compare the insights from the technical efficiency analysis presented in the main part of the paper to an ANCOVA analysis that is commonly used in similar studies. Table 7 reports the descriptive statistics (mean and standard deviation) of the accuracy scores and response times for the five executive function tasks, including several derived measures such as: i) the absolute difference between the incongruent and congruent trials (Difference); ii) a simple average performance measure of the congruent and incongruent trials (Average); iii) the Local Shifting Cost (LSC) and Global Shifting Cost (GSC); iv) the Inhibition effect. For all executive function measures, a series of between-groups ANCOVA analyses are performed with age, non-verbal intellectual ability, grammar skill, expressive vocabulary skill, receptive vocabulary skill, SES, and language use as covariates.

**Table 7:** Comparison with conventional designs – ANCOVA analysis

Executive function task	Units	Bilinguals (*n* = 32)		Greek monolinguals (*n* = 38)		ANCOVA	
		Mean	*SD*	Mean	*SD*	F-stat	η^2^
Backward digit span (ACC)	%	0.624	0.097	0.668	0.080	6.50^**^	0.096
Backward digit span (RT)	ms	933.30	289.95	807.11	271.59	0.49	0.008
Counting recall (ACC)	%	0.494	0.144	0.478	0.139	4.32^**^	0.066
Counting recall (RT)	ms	2394.90	1215.61	1887.87	1179.38	0.00	0.000
Colour shape (ACCcong)	%	0.918	0.068	0.904	0.072	0.54	0.009
Colour shape (ACCincong)	%	0.725	0.124	0.756	0.123	0.07	0.001
Colour shape (ACCincong-cong)	%	0.193	0.113	0.148	0.104	0.02	0.000
Colour shape (ACCaverage)	%	0.821	0.083	0.830	0.086	0.26	0.004
Colour shape (RTcong)	ms	786.68	190.01	843.95	170.05	1.68	0.027
Colour shape (RTincong)	ms	1056.67	219.40	1081.82	166.10	1.57	0.025
Colour shape (RTincong-cong)	ms	284.69	145.37	259.95	130.90	0.15	0.002
Colour shape (RTaverage)	ms	921.67	186.50	962.89	147.44	2.23	0.035
LSC	ms	– 161.33	142.34	-134.70	161.24	0.15	0.002
GSC	ms	– 376.43	226.90	-330.86	209.02	0.06	0.001
Stroop (ACCcong)	%	0.935	0.104	0.929	0.101	1.37	0.022
Stroop (ACCincong)	%	0.825	0.231	0.824	0.211	0.02	0.000
Stroop (ACCbase)	%	0.921	0.095	0.948	0.066	3.55^*^	0.055
Stroop (ACCincong-cong)	%	0.154	0.207	0.115	0.160	0.66	0.011
Stroop (ACCaverage)	%	0.894	0.117	0.900	0.109	0.90	0.014
Stroop (RTcong)	ms	762.14	302.45	684.87	196.91	0.69	0.011
Stroop (RTincong)	ms	1027.67	333.47	958.86	308.02	0.29	0.005
Stroop (RTbase)	ms	812.15	304.88	718.23	185.53	0.96	0.015
Stroop (RTincong-cong)	ms	277.30	165.94	284.74	171.68	0.00	0.000
Stroop (RTaverage)	ms	861.46	298.93	784.92	195.46	1.41	0.023
Inhibition effect	ms	– 265.53	184.75	– 274.00	188.78	0.00	0.000
ANT (ACCcong)	%	0.949	0.068	0.970	0.048	0.75	0.012
ANT (ACCincong)	%	0.913	0.114	0.938	0.103	0.15	0.002
ANT (ACCincong-cong)	%	0.042	0.058	0.036	0.088	0.05	0.001
ANT (ACCaverage)	%	0.931	0.089	0.954	0.067	0.38	0.006
ANT (RTcong)	ms	863.15	243.77	824.74	182.19	2.71	0.043
ANT (RTincong)	ms	964.44	273.86	902.51	206.10	1.87	0.030
ANT (RTincong-cong)	ms	102.17	67.56	81.56	57.50	0.16	0.003
ANT (RTaverage)	ms	912.30	255.63	861.81	189.42	2.40	0.038

*Notes.* The table reports key means and standard deviations (*SD*) for the executive functions test of the bilingual and monolinguals groups. *n* denotes the sample size, ACC denotes the accuracy score, RT the response time in msec. “cong” and “incong” refer to the congruent and incongruent respectively; “incong-cong” is the absolute difference between the incongruent and congruent trials; “average” is the average of the congruent, incongruent, and base trials. Local switching cost (LSC), Global switching cost (GSC) and Inhibition effect are explained in “Executive function tasks” section). F-stat and η^2^ correspond to the between-subjects ANCOVA analysis with age, non-verbal intellectual ability, grammar score, language score, vocabulary score, SES and language use as covariates. ***, **, * denote statistical significance at the 1, 5, and 10 significance level, respectively

The results of this analysis suggest that there are no significant performance differences between bilingual and monolinguals. For example, and pertaining to the working memory, no conclusive significant difference is found with bilinguals performing better in the Count recall task (*F*(1, 61) = 4.32, *p* < .05), but worse in the BDST (*F*(1, 61) = 6.50, *p* < .05) compared to their monolingual counterparts. However, a drawback of an ANCOVA analysis is apparent in this case as it is not able to account for the multiple executive function tasks (and their metrics) that are available. As an alternative, we use a MANCOVA analysis that allows for multiple dependent variables at the same time, thereby allowing for more efficient use of the breadth of the administered executive function tests. We use the same control variables as in the ANCOVA case. With regards to the choice of the dependent variables, we present a list of several models, labelled I–X, in Table 8, each using different metrics of each executive function score. In the MANCOVA models presented, we include at least one dependent variable from each of the three categories of executive function, namely working memory, switching and inhibition. These results are reported in Table 8.

**Table 8:** Comparison with conventional designs – MANCOVA analysis

Executive function measures	I	II	III	IV	V	VI	VII	VIII	IX	X
Backward digit span (ACC)	YES	YES		YES	YES	YES	YES	YES	YES	YES
Backward digit span (RT)			YES				YES	YES	YES	YES
Counting recall (ACC)	YES	YES			YES	YES	YES	YES	YES	YES
Counting recall (RT)			YES	YES			YES	YES	YES	YES
Colour shape (ACCaverage)	YES								YES	YES
Colour shape (RTaverage)			YES	YES					YES	YES
LSC						YES	YES			
GSC		YES			YES			YES		
Stroop (ACCaverage)	YES								YES	YES
Stroop (RTaverage)			YES	YES					YES	YES
Inhibition effect		YES			YES	YES	YES	YES		
ANT (ACCaverage)	YES				YES	YES	YES	YES	YES	YES
ANT (RTaverage)		YES	YES	YES			YES	YES	YES	YES
Controls										
Age	YES	YES	YES	YES	YES	YES	YES	YES	YES	YES
IQ	YES	YES	YES	YES	YES	YES	YES	YES	YES	YES
GS, LS, VS	YES	YES	YES	YES	YES	YES	YES	YES	YES	YES
SES	YES	YES	YES	YES	YES	YES	YES	YES	YES	NO
Language use	YES	YES	YES	YES	YES	YES	YES	YES	YES	NO
Observations	70	70	70	70	70	70	70	70	70	70
Wilks Lambda	0.830	0.827	0.867	0.817	0.828	0.828	0.806	0.803	0.754	0.710
F-statistic	2.340^*^	2.380^**^	1.750	2.550^*^	2.380^**^	2.360^*^	1.630	1.660	1.690	2.200^**^

*Notes.* The table reports ten MANCOVA models (labelled I–X) where the dependent variables are metrics of each executive function score. “YES” denotes which dependent variables are used in each model. “average” is the average of the congruent, incongruent, and base trials. Local switching cost (LSC), Global switching cost (GSC) and Inhibition effect are explained in “Executive function tasks” section). Each MANCOVA model controls for age, non-verbal intellectual ability, grammar score (GS), language score (LS), vocabulary score (VS), SES, and language use. The Wilks’ Lambda and associated F-statistic relate to the between-groups comparison. ^***^, ^**^, ^*^ denote statistical significance at the 1, 5, and 10% significance level

Overall, the MANCOVA results suggest that there are significant differences between the two groups. For example, under Model I, the between-group tests suggest significant differences in the executive function of the two groups (*F*(1, 61) = 2.34, *p* < .10; Wilk’s Λ = 0.830). Model X is of particular interest by featuring as dependent variables the same measures used in the technical efficiency analysis as outputs, while the controls variables correspond to the inputs. This model suggests of significant between-group differences in executive function (*F*(1, 63) = 2.20, *p* < .05; Wilk’s Λ = 0.710). Hence, the qualitative conclusion obtained using the technical efficiency approach is verified by a MANCOVA analysis. A drawback of the MANCOVA compared to technical efficiency is that subsequent analysis in the former case is more complex, as between-group marginal effects are unique in each dependent variable.

### Comparison to an alternative dataset - the Antoniou et al. (2016) dataset

The effect of bilectalism and multilingualism on executive control is examined in Antoniou et al. (2016). In this section, we revisit the Antoniou et al. (2016) dataset, and apply the technical efficiency approach in answering the same questions. The use of the particular dataset is motivated from the conceptual closeness of the investigated topic – i.e., executive function in bilingual/bilectal populations, as well as the number of administered executive function tasks coupled with the identified need to arrive to a comprehensive measure that summarises all information. In particular, the authors administer six executive function tasks on a sample of bilectal, multilingual, and monolingual children. Subsequently, they use a principal component analysis (PCA) technique to produce two composite measures, which they identify as representative of working memory and inhibition. The executive function tasks in Antoniou et al. (2016) are the following: i) Backward digit span (BDST); ii) Corsi blocks forward (Corsi forward); iii) Corsi blocks backward (Corsi backward); iv) Soccer task (Stroop); v) Simon task; vi) Colour shape. For these tasks, the dataset provides either the number of correct trials (BDST, Corsi) or accuracy scores and response times (Soccer, Simon, Colour shape).

We apply the technical efficiency methodology as described in the main paper with the outputs being the percentage scores of each of the three working memory tasks (BDST, Corsi forward, Corsi backward), and the accuracy scores of the Soccer, Simon, and Colour shape tasks. Our choice of inputs is similarly motivated to our main analysis but also takes into account the availability of the data. In particular, we use three inputs: i) non-verbal intellectual ability (IQ), ii) general language ability, iii) vocabulary skill (PPVT), iv) age. We conduct our analyses on three samples, labelled S1-S3. The first (S1) compares bilectal and monolingual children, while the second (S2) compares bilectal, multilingual, and monolingual children. These two use the exact sample specifications of Antoniou et al. (2016) for a direct comparison.

In particular, there are 17 bilectal participants (*M* = 7.6 years of age; *SD* = 0.9 years) that are speakers of Cypriot Greek and Standard Modern Greek, while the 25 monolingual participants (*M* = 7.4 years of age; *SD* = 0.9 years) only speak Standard Modern Greek under S1. The background analysis in Antoniou et al. (2016) suggests that these two groups do not differ in age, gender, or language comprehension, however the bilectals exhibit significantly lower expressive and receptive vocabulary scores. Under S2, there are 44 bilectal participants (*M* = 7.6 years of age; *SD* = 0.9 years), 26 multilingual participants (*M* = 7.6 years of age; *SD* = 0.9 years) and 25 monolingual participants (*M* = 7.4 years of age; *SD* = 0.9 years). The background analysis in Antoniou et al. (2016) suggests that these three groups do not exhibit significant differences in age, gender, or language comprehension, however there are significant differences in terms of SES and IQ. Our third (S3) analysis compares bilectal, multilingual, and monolingual children and this time we use all the participants that are available in the Antoniou et al. (2016) dataset. Under S3, there are 64 bilectal participants (*M =* 7.8 years of age; *SD =* 1.59 years), 47 multilingual participants (*M =* 7.8 years of age; *SD =* 1.8 years) and 25 monolingual participants (*M =* 7.6 years of age; *SD =* 0.9 years). These three groups do not exhibit statistically significant differences with respect to age (*F*(2, 133) = 0.550, *p* > .10), gender (*F*(2, 133) = 0.370, *p* > .10), and IQ (*F*(2, 130) = 2.270, *p* > .10). There are significant differences in terms of SES (*F*(2, 130) = 10.43, *p* < .01) and general language ability (*F*(2, 133) = 6.830, *p* < .01).

Table 9, panels A–C present the results of this analyses of S1–S3, respectively. In each group, we report the mean and standard deviation of the technical efficiency as well as the working memory and inhibition composite measures of Antoniou et al. (2016) for comparison purposes. An ANCOVA between-groups analysis is reported with age, IQ, general language ability, and SES as control variables, in line with those used in Antoniou et al. (2016).

**Table 9:** Comparison to alternative datasets – the Antoniou et al. (2016) dataset

Panel A: Sample 1								
	Bilectals (*n* = 17)				Monolinguals (*n* = 25)			
	Mean	*SD*			Mean	*SD*	F-statistic	
Working memory	0.039	0.653			– 0.289	0.763	2.960^*^	
Inhibition	0.123	0.730			– 0.098	0.557	1.140	
Technical Efficiency	0.988	0.031			0.955	0.057	3.120^*^	
t-test	2.121^**^							
Panel B: Sample 2								
	Bilectals (*n* = 44)		Multilinguals (*n* = 26)		Monolinguals (*n* = 25)			
	Mean	*SD*	Mean	*SD*	Mean	*SD*	F-statistic	
Working memory	– 0.033	0.712	0.208	0.658	– 0.289	0.763	2.630^*^	
Inhibition	0.126	0.561	0.198	0.465	– 0.098	0.557	1.640	
Technical Efficiency	0.950	0.090	0.979	0.035	0.896	0.091	7.010^***^	
t-statistic	2.401^**^		4.308^***^					
Panel C: Sample 3								
	Bilectals (*n* = 64)		Multilinguals (*n* = 47)		Monolinguals (*n* = 25)			
	Mean	*SD*	Mean	*SD*	Mean	*SD*	F-statistic	
Working memory	– 0.027	0.800	0.036	0.882	– 0.289	0.763	1.060	
Inhibition	0.021	0.622	– 0.004	0.675	– 0.098	0.557	0.620	
Technical Efficiency	0.950	0.089	0.949	0.146	0.842	0.124	6.100^***^	
t-statistic	4.595^***^		3.118^***^					

*Notes.* The table reports mean and standard deviation (*SD*) of the technical efficiency estimates in each of the bilectals, multilinguals, and monolingual groups using the dataset of Antoniou et al. (2016), where *n* denotes the sample size. The working memory and inhibition are the composite scores as these are defined in Antoniou et al., (2016) and are reported here for comparison purposes. Panel A compares the bilectals to the monolinguals, while panel B compares bilectals, multilinguals, and monolinguals. These two use the exact sample specifications of Antoniou et al. (2016). Panel C compares bilectals, multilinguals, and monolinguals using a more extended dataset. The ANCOVA F-statistic presented is for the group categorical variable, where a statistically significant difference between the respective groups is indicated. The covariates used in the ANCOVA are: age, IQ, general language ability and SES. The *t*-statistic is for the between-groups mean comparison test of the technical efficiency of the respective group to the monolingual. ***, **, * denote statistical significance at the 1, 5, and 10% significance levels

For S1, the technical efficiency analysis shows the bilectals to be about 3.2 percentage points more efficient than their monolingual counterparts with the difference between the groups being significant (*F*(1,36) = 4.53, *p* < .05). Antoniou et al. (2016) use a 2x2 mixed ANCOVA design for the working memory and inhibition components and find the bilectals to outperform the monolinguals.

The technical efficiency analysis on S2 uncovers significant differences between the groups (*F*(2,88) = 7.01, *p* < .01). Specifically, the multilinguals are the most efficient group with an average efficiency score of 0.979, followed by the bilectals at 0.95 and the monolinguals at 0.89. The difference between multilinguals and monolinguals is significant (*F*(1,45) = 10.21, *p* < .01), while a similar conclusion is reached for bilectals and monolinguals (*F*(1,63) = 8.91, *p* < .01). No significant difference is found between the bilectals and the multilinguals (*F*(1,64) = 1.40, *p* > .10). Antoniou et al. (2016) use a 2x3 mixed ANCOVA design and find that bilectals and multilinguals significantly outperform the monolingual group in terms of executive function. However, no significant difference between the bilectals and multilinguals is observed.

The technical efficiency analysis on S3 shows a similar conclusion to S2 with both multilinguals and bilectals being more efficient than their monolingual counterparts. The between-groups ANCOVA analysis suggests that the difference is statistically significant (*F*(2, 129) = 6.10, *p* < .01). A 2x3 (working memory versus inhibition by group: multilinguals versus bilectals versus monolinguals) ANCOVA, in the spirit of Antoniou et al. (2016), does not suggest any significant difference in the three groups (*F*(2, 121) = 1.145, *p* > .10).

Overall, we confirm the results of the Antoniou et al. (2016) using our technical efficiency approach and offer some more insights in terms of the main advantages of a technical efficiency approach. Compared to the PCA, technical efficiency provides a single ratio, which ranges by construction between 0 and unity, and has a clear interpretation. By contrast, the PCA requires a degree of subjectivity in terms of the number of retained components (or factors), with the Kaiser’s criterion being one of the many used in such analysis (Antoniou et al., 2016). An inherent difficulty in the PCA related to the interpretation of the factors. Another advantage of technical efficiency is that by construction it accounts for differences between the groups in the form of inputs. For example, the PCA analysis is followed by an ANCOVA that accounts for certain differences between the two groups in several metrics. By contrast, several of these metrics may be used as inputs in the technical efficiency analysis. As a consequence, simple unconditional *t* tests on the technical efficiency estimates have certain merit. An inspection of the *t* statistics reported in Table 9 yields the same qualitative conclusion as the more complex ANCOVA setup.^20^

### The balanced bilinguals assumption

In the main analysis when creating the grammar, receptive, and expressive vocabulary scores for the bilinguals we used what we termed as composite score 2 (see “Second stage analysis” section for more details). Here we compare to the naïve and restrictive strategy where the main assumption is that bilinguals are balanced between the two languages, namely Greek and English. As a consequence, the composite grammar skill score would be a simple average of the respective grammar skill tasks for Greek and English languages (CS1). Admittedly this may seem a strong assumption particularly in cases where some participants may have had limited exposure to the new language. However, as this strategy is less computationally demanding, there is a certain merit in examining the impact of the results from adopting it.

Table 10 replicates Table 5 with the only difference being that bilinguals are now assumed to be balanced using the composite score we explain above. A cursory inspection of the results suggests that the qualitative nature of the story holds, with bilinguals having higher technical efficiency than their monolingual counterparts. However, this gap in efficiency appears less pronounced compared to our main analysis. In particular, for the overall results, bilinguals are now about 11.6% more efficient. Hence, the assumption of balanced bilingualism in this instance reduces the efficiency advantage of the bilinguals by approximately 10% compared to the main analysis (see Table 5). Individual executive function tasks show higher variability. For instance, the bilinguals in the BDST task show a 17.7% lower gain in their efficiency scores to the monolinguals compared to the results of Table 5. Overall, the implicit assumption of balanced bilinguals that appears in the calculation of the composite scores has an important effect.

**Table 10:** Technical efficiency estimates by group – balanced bilinguals assumption

	Monolinguals			Bilinguals			Mean % gain	Anova F-test	T-test	MW-test	KS-test
Executive function task	Mean	*SD*	Median	Mean	*SD*	Median					
Panel A											
Backward digit span	0.754	0.108	0.736	0.821	0.147	0.834	8.58	4.87^**^	2.21^**^	2.37^**^	0.34^**^
Counting recall	0.644	0.092	0.620	0.742	0.145	0.709	14.12	11.75^***^	3.42^***^	3.33^***^	0.48^***^
Colour shape	0.649	0.143	0.655	0.782	0.164	0.761	18.62	13.10^***^	3.62^***^	3.06^***^	0.36^**^
Non-verbal Stroop	0.637	0.126	0.613	0.738	0.157	0.727	14.82	9.05^***^	3.01^***^	2.97^***^	0.48^***^
ANT	0.618	0.112	0.600	0.724	0.162	0.707	15.82	10.37^***^	3.22^***^	2.97^***^	0.43^***^
Total	0.660	0.116	0.645	0.762	0.155	0.748	14.25				
Panel B											
Accuracy	0.790	0.101	0.766	0.879	0.118	0.904	10.65	11.53^***^	3.39^***^	3.08^***^	0.41^***^
Response speed	0.580	0.159	0.547	0.692	0.222	0.689	17.64	6.00^**^	2.44^**^	2.28^**^	0.32^*^
All	0.793	0.100	0.770	0.891	0.121	0.942	11.62	13.74^***^	3.70^***^	3.35^***^	0.44^***^

*Notes.* The table presents DEA technical efficiency estimates for the monolingual and bilingual groups of children of our sample. The outputs in each executive function task are: i) accuracy, and ii) response speed. The outputs of all five executive function tasks are utilised in the “All” variant. The “accuracy” and “response speed” variants use the accuracy scores and response speed scores of all executive function tasks, respectively. Five inputs are utilised, namely: i) non-verbal intellectual ability, ii) grammar skill, iii) expressive vocabulary skill, iv) receptive vocabulary skill, v) age. The weighting scheme for the bilingual inputs is based on Composite score 1 (see “Second stage analysis” section). For each group we present the mean, standard deviation, and median of technical efficiency, the logarithmic percentage gain where a positive value indicates that bilinguals are more efficient than monolinguals. A battery of tests is presented including an ANOVA F-test and a bootstrap *t* test for the equality of means between the two groups, a Mann–Whitney (MW) test for the equality of medians between the two groups, a Kolmogorov–Smirnov (KS) test for the equality of the distribution of efficiency scores in the two groups. ^***^, ^**^, ^*^ denote statistical significance at the 1, 5, and 10% significance levels, respectively

## Conclusions

In this paper, we introduce a novel approach to evaluate performance in the executive functioning skills of bilingual and monolingual children. This approach is based on the frontier methodology that measures the relative efficiency of a decision-making unit (DMU) compared to the best practice, in what is termed as *technical efficiency*. Technical efficiency may be viewed as a composite performance indicator, which combines information from multiple indicators, represented by inputs and outputs, over a set of decision-making units (DMUs). Technical efficiency estimates are obtained via DEA and are used to benchmark the DMUs, with the efficient DMUs described as “best-practice”. Hence, it is particularly useful in performance evaluation situations where there are several alternative metrics. It is worth pointing out that an efficient DMU has the best composite performance (i.e., is technically efficient) using all the available information reflected in inputs and outputs. By contrast, the complex nature of executive function may be insufficiently captured by analysing single metrics in isolation; often leading to mixed conclusions. An alternative approach might be to construct a weighted average of several metrics. However, an issue here is that an assumption on the weighting scheme would be needed. An additional challenge is when different measurement units are present across the metrics. By contrast, DEA optimally selects the weights thereby letting the data speak for themselves, while it can handle a variety of data subject to only two restrictions. First, DEA applications require that the factors only appear either as input or output. While this is clearly visible in the case of raw data, ratios may be more challenging if for example inputs and outputs share a common denominator. Subject to the above rule, DEA can accommodate both raw data and ratios in inputs/outputs (Cook et al., 2014; Cooper et al., 2000; Dyson et al., 2001). Second, all outputs need to be quantities where “more-the-better” is applicable; the converse is true for the inputs. In our research, the executive function tests’ accuracy and response time is an example where a transformation is required to ensure this condition is met. Technical efficiency brings several important benefits to the discipline. Most importantly, it can take into account multiple tasks and multiple metrics, which define the outputs. By construction, it accounts for differences with respect to key covariates, dubbed as inputs. Being a non-parametric, linear programming technique means that it is flexible, does not rely on distributional assumptions, and is not computationally intensive.

We demonstrate the application of the frontier methodology in the context of bilingualism, by focusing on executive function tasks in 32 Greek–English bilingual children that are compared against 38 Greek monolingual children. Using the accuracy and response times of five executive function tasks spanning working memory, inhibition, and shifting, we find the bilingual cohort to be around 6.5% more efficient compared to the Greek monolinguals, which is a statistically significant difference. This suggests that the bilinguals outperform their monolingual counterparts in terms of executive function, after controlling for differences in terms of age, non-verbal intellectual ability, grammar skill, expressive vocabulary skill, receptive vocabulary skill, SES, and language use. The results are robust to a number of alternative specifications of technical efficiency (e.g., using only the accuracy metric), alternative specification of control variables (e.g., with/-out SES, language use), estimation techniques (e.g., ANCOVA, bootstrap regression, k-means nearest neighbours). To identify the benefits of technical efficiency analysis, we subject our dataset to a conventional ANCOVA / MANCOVA series of analyses. The ANCOVA suggests no distinct evidence of a bilingual superior performance, across a wide range of metrics that are in line with the recent literature. However, the MANCOVA approach owing to its multivariate nature, is able to pick up differences between the two groups. In particular, the MANCOVA and the technical efficiency with the same dependent variables are able to provide similar results; thus, highlighting the merits of technical efficiency. We also apply the technical efficiency approach to an alternative, yet related, dataset sourced from Antoniou et al. (2016). Using our technical efficiency approach we are able to replicate the qualitative conclusions of the Antoniou et al. (2016), which uses principal component analysis. We also comment on the advantages of technical efficiency relatively to principal component analysis; namely the more intuitive nature of the efficiency score, and the fact that it controls by construction for several differences between the two groups. Future research may incorporate technical efficiency analysis along the lines outlined here, expand into more tasks that would cover additional aspects of executive function.

## Data Availability

The data and code generated during the current study are available at: https://bangoroffice365-my.sharepoint.com/:f:/g/personal/elp4ae_bangor_ac_uk/ErsUByMXntFBobeDz60SVpQB1kZBqVzcjLyRb-2ggf6UTw?e=VoYPJ0

## Appendix 1

**Table 11:** Correlation matrix

	BDST_ACC	Counting_ACC	Colour shape_ACC	Stroop_ACC	ANT_ACC	BDST_RT	Counting_RT	Colour shape_RT	Stroop_RT	ANT_RT	SES	IQ	GS	LS	VS
Counting_ACC	0.46	1.00													
	(0.00)														
Colour shape_ACC	0.33	0.55	1.00												
	(0.01)	(0.00)													
Stroop_ACC	0.57	0.45	0.45	1.00											
	(0.00)	(0.00)	(0.00)												
ANT_ACC	0.52	0.52	0.56	0.65	1.00										
	(0.00)	(0.00)	(0.00)	(0.00)											
BDST_RT	– 0.04	– 0.06	– 0.04	– 0.05	– 0.01	1.00									
	(0.73)	(0.62)	(0.72)	(0.70)	(0.94)										
Counting_RT	– 0.24	– 0.09	– 0.27	– 0.42	– 0.48	0.02	1.00								
	(0.05)	(0.46)	(0.02)	(0.00)	(0.00)	(0.89)									
Colour shape_RT	– 0.30	– 0.37	– 0.20	– 0.33	– 0.45	0.11	0.28	1.00							
	(0.01)	(0.00)	(0.10)	(0.01)	(0.00)	(0.36)	(0.02)								
Stroop_RT	– 0.46	– 0.51	– 0.38	– 0.41	– 0.54	0.11	0.31	0.57	1.00						
	(0.00)	(0.00)	(0.00)	(0.00)	(0.00)	(0.38)	(0.01)	(0.00)							
ANT_RT	– 0.58	– 0.41	– 0.40	– 0.51	– 0.65	0.19	0.38	0.61	0.64	1.00					
	(0.00)	(0.00)	(0.00)	(0.00)	(0.00)	(0.12)	(0.00)	(0.00)	(0.00)						
SES	– 0.07	– 0.07	0.05	– 0.03	0.04	0.08	0.16	– 0.04	0.08	0.02	1.00				
	(0.55)	(0.56)	(0.67)	(0.82)	(0.73)	(0.50)	(0.18)	(0.76)	(0.53)	(0.90)					
IQ	0.61	0.68	0.39	0.45	0.59	– 0.06	– 0.19	– 0.54	– 0.59	– 0.61	0.00	1.00			
	(0.00)	(0.00)	(0.00)	(0.00)	(0.00)	(0.64)	(0.11)	(0.00)	(0.00)	(0.00)	(1.00)				
GS	0.41	0.49	0.41	0.42	0.43	– 0.03	– 0.21	– 0.46	– 0.43	– 0.47	– 0.01	0.57	1.00		
	(0.00)	(0.00)	(0.00)	(0.00)	(0.00)	(0.80)	(0.08)	(0.00)	(0.00)	(0.00)	(0.94)	(0.00)			
LS	0.25	0.26	0.32	0.22	0.26	0.04	– 0.11	– 0.35	– 0.26	– 0.27	0.16	0.38	0.40	1.00	
	(0.03)	(0.03)	(0.01)	(0.06)	(0.03)	(0.74)	(0.37)	(0.00)	(0.03)	(0.03)	(0.18)	(0.00)	(0.00)		
VS	0.52	0.54	0.52	0.35	0.53	0.07	– 0.27	– 0.51	– 0.52	– 0.51	0.04	0.67	0.64	0.61	1.00
	(0.00)	(0.00)	(0.00)	(0.00)	(0.00)	(0.57)	(0.03)	(0.00)	(0.00)	(0.00)	(0.74)	(0.00)	(0.00)	(0.00)	
Age	0.57	0.62	0.46	0.48	0.63	– 0.12	– 0.41	– 0.66	– 0.73	– 0.71	– 0.17	0.78	0.58	0.45	0.72
	(0.00)	(0.00)	(0.00)	(0.00)	(0.00)	(0.32)	(0.00)	(0.00)	(0.00)	(0.00)	(0.16)	(0.00)	(0.00)	(0.00)	(0.00)

*Notes.* The table reports correlation coefficients and *p* values in parentheses. BDST denotes the Backward digit span task, ACC denotes accuracy score, RT the response time. GS, LS, VS, are the grammar score, language score, and vocabulary score (VS), respectively. SES is the socio-economic status

## Technical Appendix 2

*The k-means nearest-neighbour matching*

The k-means nearest-neighbour matching relies on some distance function to quantify the closeness between two (or more) observations. In our context, for each observation of a bilingual child, the k-means nearest-neighbour approach determines the “nearest” observation of a monolingual.

A distance function is used to define the closeness of the observations. In the general form we can denote this variable as *x*. Then the distance between two observations *i*, *j* where the *i* observations correspond to a bilingual and the *j* observation corresponds to a monolingual is given as:

5 $$ \left|{x}_i-{x}_j\right|=\frac{\left({x}_i-{x}_j\right)\left({x}_i-{x}_j\right)}{Cov\left(x,x\right)} $$

We can generalise this formula for *p* number of covariates using matrix algebra. Assume that *x* = {*x*_1_, *x*_2_, …, *x*_*p*_} and that each observation, *i*, has the following set of covariates **x**_*i*_ = {*x*_1, *i*_, *x*_2, *i*_, …, *x*_*p*, *i*_}. The distance between observations *i*, *j* is now given as:

6 $$ \left\Vert {\mathbf{x}}_i-{\mathbf{x}}_j\right\Vert ={\left({\left({\mathbf{x}}_i-{\mathbf{x}}_j\right)}^{\prime }{\mathbf{S}}^{-1}\left({\mathbf{x}}_i-{\mathbf{x}}_j\right)\right)}^{1/2} $$

where **S** is the variance-covariance matrix of the covariates.

Typical choices for ***S*** are:

7 $$ \mathbf{S}=\left\{\begin{array}{c}{\mathbf{I}}_{\boldsymbol{p}}\kern0.75em \mathrm{for}\ \mathrm{the}\ \mathrm{Euclidean}\ \mathrm{case}\\ {}\frac{\left(\mathbf{X}-\overline{\mathbf{x}}^{\prime }{\mathbf{1}}_{\boldsymbol{n}}\right)^{\prime}\boldsymbol{W}\left(\mathbf{X}-\overline{\mathbf{x}}^{\prime }{\mathbf{1}}_{\boldsymbol{n}}\right)}{\sum_i^n{w}_i-1}\kern0.75em \mathrm{for}\ \mathrm{the}\ \mathrm{Mahalanobis}\ \mathrm{case}\end{array}\right. $$

where **1**_***n***_ is an *n* × 1 vector of ones, **I**_***p***_ is the identity matrix of order *p*, same as the number of covariates used, *w*_*i*_ is the frequency weight for the *i* observation, $\overline{\mathrm{x}}={\sum}_i^n{w}_i{\mathrm{x}}_i/{\sum}_i^n{w}_i$ denotes a weighted mean and **W** is an *n* × *n* diagonal matrix containing the frequency weights. Compared to the Euclidean case, the Mahalanobis may be preferred as it accounts for interactions between the covariates.

Coming back to observation *i*, we can define the following set of nearest-neighbour index:

8 $$ \varOmega {(i)}^x=\left\{j|{t}_j=1-{t}_i,\left\Vert {\mathbf{x}}_i-{\mathbf{x}}_j\right\Vert <{\left\Vert {\mathbf{x}}_i-{\mathbf{x}}_l\right\Vert}_{\mathbf{S}},{t}_l=1-{t}_i\forall l\ne j\right\} $$

where *i* is the observation corresponding to a bilingual and for which we want to find a matching monolingual. *j* denotes the matching monolingual (is only one in this case) and *l* denotes another candidate monolingual. *t* denotes the treatment effect and takes the value 1 for bilinguals, zero otherwise. ‖**x**_*i*_ − **x**_*j*_‖ and ‖**x**_*i*_ − **x**_*l*_‖ denote the distance between *i*, *j* and *i*, *l*, respectively, and in the formula above we require that the distance between *i*, *j* is smaller than *i*, *l* (since we select the matching *j* participant as our match). The notation *t*_*j*_ = 1 − *t*_*i*_ and *t*_*l*_ = 1 − *t*_*i*_ implies that for our *i* observation which is a bilingual (hence *t*_*i*_ = 1) needs to be matched with some monolingual observation for which *t*_*j*_ = 1 − 1 = 0 or *t*_*l*_ = 1 − 1 = 0

The above can be generalised for *m* matching observations to enhance reliability of the comparisons, as follows:

9 $$ \varOmega {(i)}_m^x=\left\{{j}_1,{j}_2,\dots, {j}_m|{t}_{j_k}=1-{t}_i,{\left\Vert {\mathbf{x}}_i-{\mathbf{x}}_{j_k}\right\Vert}_{\mathbf{S}}<{\left\Vert {\mathbf{x}}_i-{\mathbf{x}}_{j_k}\right\Vert}_{\mathbf{S}},{t}_l=1-{t}_i\forall l\ne {j}_k\right\} $$

For the prediction of the potential outcomes, we use the following notation. *y*_1, *i*_ is the potential outcome of the *i* observation that corresponds to a bilingual (*t* = 1). Conversely, *y*_0, *i*_ is the potential outcome of the *i* observation that corresponds to a monolingual (*t* = 0). Only *y*_1, *i*_ or *y*_0, *i*_ is observed, never both. The *k*-means nearest neighbours can predict the potential outcome for the *i* observation as follows:

10 $$ {\hat{y}}_{t,i}=\left\{\begin{array}{c}{y}_i\kern0.5em \mathrm{if}\ {t}_i=t\kern0.75em \mathrm{for}\ t\in \left\{0,1\right\}\\ {}\frac{\sum_{j\in \varOmega (i)}{w}_j{y}_j}{\sum_{j\in \varOmega (i)}{w}_j}\end{array}\right. $$

The first is the case where the outcome of the individual observation (*y*_*i*_) is observed whether bilingual (*t* = 1) or monolingual (*t* = 0). The second case is the *counterfactual* outcome, which does not exist and is estimated as the outcome of the closest match (or matches). The following quantities of interest, namely the Average Treatment Effect (ATE) and the Average Treatment Effect on the Treated (ATET) can be defined as:

11 $$ ATE={\tau}_1=E\left({y}_1-{y}_0\right) $$

12 $$ ATET={\delta}_1=E\left({y}_1-{y}_0|t=1\right) $$

## Footnotes

1 The comparison of bilinguals and monolinguals is not exhausted within the executive function literature, see for example Hartsuiker et al. (2004) for a comparison in terms of lexical and syntactic information and Bialystok et al. (2015) for an investigation of how bilingualism affects particular aspects of the languages used.

2 In other contexts, and within the standard econometrics literature, this would amount to omitted variables bias (Greene, 2003).

3 No clear definition on the number of participants of a large sample size exists; however all cited studies feature at least 230 participants, while Paap et al. (2014) suggest that participants in each group should be at least 180.

4 Paap and Greenberg (2013) identify a causality issue in this case, where it may be argued that players excel in sports *because* they exhibit higher executive function. Arguably, this may have been attributed to their growing up in a bilingual environment, and therefore some bilingual advantage been bestowed upon them. However, empirically testing this appears challenging.

5 This is not a unique problem in this research strand. In finance, a survey of 374 studies finds that a total of 56 different measures have been used to proxy firm performance (Chen et al., 2015).

6 Our sample size, a total of 70 children, is impacted by the fact that five executive function tasks are administered to all participants. As such, the sample size is larger than the study of Poulin-Dubois et al. (2011) who also administer multiple executive function tasks, but is smaller than the study of Duñabeitia et al. (2014) who administer a single executive function task on about 500 children. We use regression with bootstraped standard errors to correct for any small sample bias.

7 A few participants from either group have limited knowledge of other languages. This information is revealed to us via the parental questionnaire. The level of knowledge in these other languages is significantly inferior to the main languages under examination (i.e., English and Greek), with participants only knowing a handful of words. The level of knowledge between the Bilinguals and the Greek Monolinguals shows no significant difference between them, which suggests no potential heterogeneity induced by these participants in the analysis. As a robustness check, we exclude these participants from the analysis, and the results remain qualitatively similar.

8 We implement the DEA optimisation in LIMDEP. Other packages that have been used are Stata, R, Matlab as well as several dedicated software for DEA estimation (e.g, DEAP Frontier).

9 We also apply an alternative outlier treatment whereby we winsorise at the 1^st^ and 99^th^ percentiles of the response times within each executive function test. Using these response times does not challenge our results. For more detailed insights on data outlier treatments in the context of bilingualism, we direct you to Zhou and Krott (2016).

10 The transformation is inspired by Bayesian analysis where the inverse of standard deviation (dubbed as precision) is typically used. Other ways are also available. One would be to take the inverse of accuracy for output 1 instead. A more challenging approach would entail classifying response time as a “bad output” in line with the studies of Fukuyama and Matousek (2011). The approach of multiplying the response times by -1 (see Antoniou et al., 2016) does not work with efficiency analysis as the inputs and outputs need to be positive.

11 A robustness check with CS3 instead of CS2 is also performed with the results being qualitatively similar; hence this is not reported for brevity.

12 The data are available at: https://bangoroffice365-my.sharepoint.com/:f:/r/personal/elp4ae_bangor_ac_uk/Documents/%27The%20Executive%20Function%20of%20Bilingual%20and%20Monolingual%20Children-%20A%20Technical%20Efficiency%20Approach%27%20data%20and%20code?csf=1&web=1&e=HeKyPF

13 A few participants from either group have limited knowledge of other languages. This information is revealed to us via the parental questionnaire. The level of knowledge in these other languages is significantly inferior to the main languages under examination (i.e., English and Greek), with participants only knowing a handful of words. The level of knowledge between the Bilinguals and the Greek Monolinguals shows no significant difference between them, which suggests no potential heterogeneity induced by these participants in the analysis. As a robustness check, we exclude these participants from the analysis, and the results remain qualitatively similar.

14 We use the music binary variable (1 if a participant plays a musical instrument, zero otherwise) to proxy for unmeasured cultural differences between the groups. Other alternatives may be participation in individual/team sports and/or other extracurricular activities. However, as Western societies the English and the Greek share a similar cultural background; hence any such effect is expected to be minimal.

15 Of these seven participants that attended Greek school in Greece, two attended for 2 year, one for 2 years, three for 3 years, and one for 4 years. Our technical efficiency results remain robust to the exclusion of these seven participants, with the results of this analysis being available from the authors upon request.

16 This is in contrast to Greek pupils attending a Greek school in the UK, which follows and delivers the Greek curriculum in Greek.

17 Higher correlation between outputs and inputs tends to increase the average efficiency scores, while the within-outputs or within-inputs correlation does not have a significant effect on the average efficiency score (López et al., 2016). In addition, the efficiency of a unit based on a DEA approach is determined by the unit’s location relative to the frontier. Therefore, the *average* efficiency score is not as informative as the *relative* efficiency score between the two (or more) groups.

18 As a robustness check, we have eliminated age from the list of input and the results remain qualitatively similar. Although these are not reported for brevity, they are available from the authors upon request.

19 The marginal effects under the ANCOVA and the regression estimation techniques are the same due to the similarity of these designs (Stuart, 2010). However, we show the marginal effect under the regression column for enhanced clarity to the reader.

20 As a further robustness check, we run a second-stage analysis similar to that of “The balanced bilinguals assumption” section, using the same control variables as in the ANCOVA that is presented. The results of this analysis do not challenge the findings here and are omitted for brevity but are available from the authors upon request.
